# Light penetration-coupled photoisomerization modeling for photodeformation of diarylethene single crystal: upscaling isomerization to macroscopic deformation

**DOI:** 10.1038/s41598-017-00910-5

**Published:** 2017-04-19

**Authors:** Muyoung Kim, Jung-Hoon Yun, Maenghyo Cho

**Affiliations:** Division of Multiscale Mechanical Design, School of Mechanical and Aerospace Engineering, Seoul National University, Seoul, 08826 Republic of Korea

## Abstract

Diarylethene is one of the photo-responsive materials that show rapid and reversible changes in their color/electrochemical properties and macroscopic deformations in the crystalline phase by light irradiation. Photoisomerization is the main cause of the photo reactivity of diarylethene, and we established a statistical model based on the density matrix formalism, which predicts quantitative isomerization progress as a population term. The model reflects photo-switching properties of the target molecule, which were characterized by first principle calculations, and external stimulus factors (light irradiation conditions and temperature). By merging light penetration physics with the model, we derived light penetration depth dependent isomerization progress to theoretically investigate photodeformation of single crystal. The model well reproduced in-plane shear deformation under ultraviolet light irradiation which would provide guideline for photoactuator design. In addition, the statistical model addressed crucial findings (primary stimuli and molecular design parameter for increasing the isomerization rate, external stimuli enhancing fluorescence performance) itself.

## Introduction

**Figure 1:**
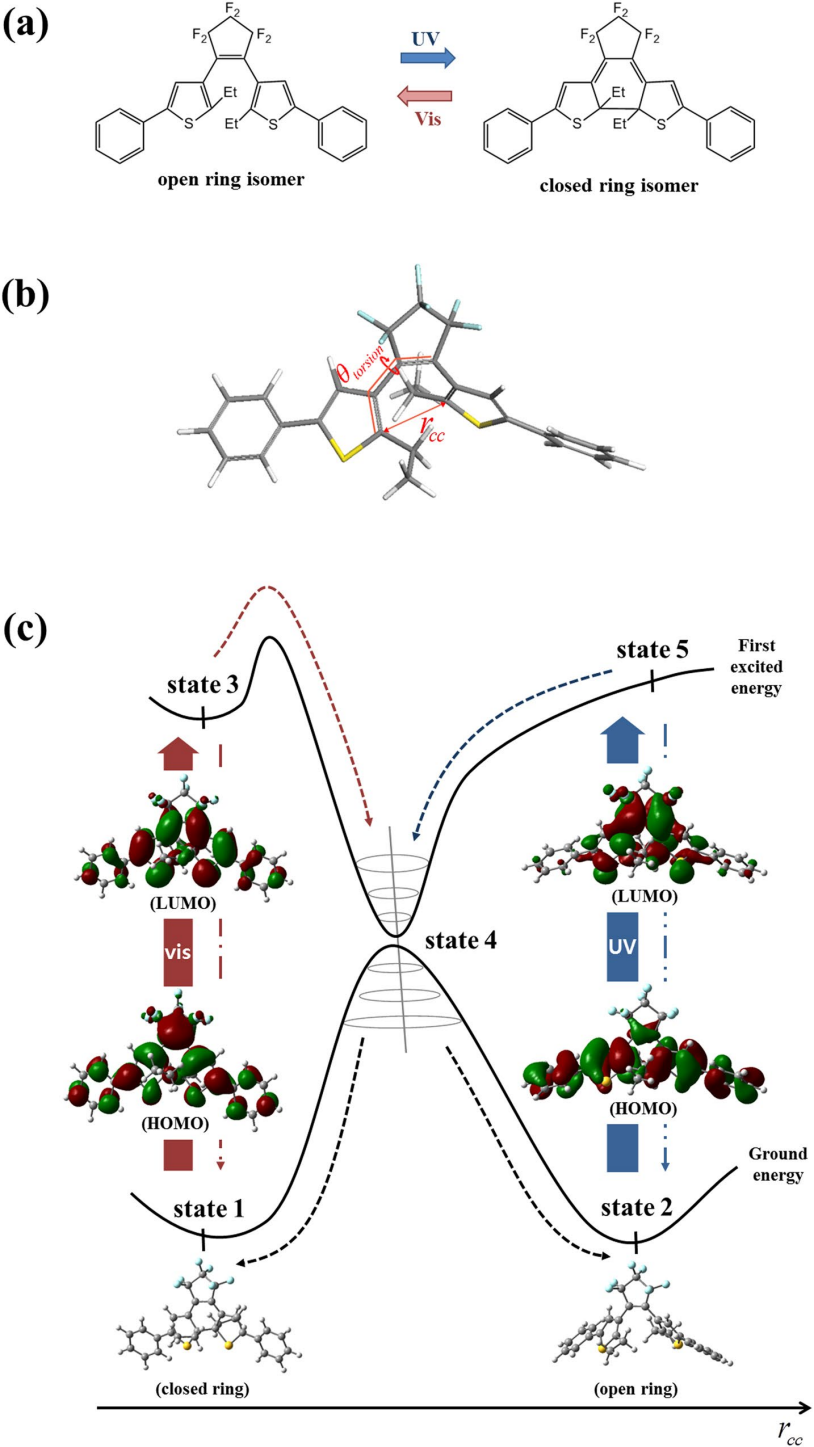
(**a**) Photoisomerization of diarylethene. (**b**) Reaction coordinate of ring opening/closing reaction. (**c**) The population transition path consists of radiative and non-radiative processes. Excitation (➖) and spontaneous decay (─·─) indicate radiative transition. Vibrational relaxation (─ ─ ─ ─) corresponds to non-radiative process. Each molecular orbital transition (HOMO → LUMO, isovalue: 0.02) corresponds to the most significant contribution for the excitation (ground- to first excited state) which is obtained from TDDFT calculation.

Among various kinds of photo-responsive materials, diarylethene is one of the most promising candidates for its thermal stability, rapid and reversible isomerization switching, and fatigue resistance^1^. Because of its potential functionality, it is regarded as a promising material that can be used in various devices (e.g. actuators, photo-optical switches, and optical memory)^1–3^. Diarylethene in the crystalline phase has received much attention for its macroscopic deformation, and changes in the color and surface morphology by light irradiation^4–9^. The fundamental physics of its photo reactivity lies with the photoisomerization, which closes the open-ring molecule under ultraviolet (UV) light, and opens the closed-ring molecule when irradiated with visible (vis) light (Fig. 1a). As photoisomerization changes the molecular volume and the changed volume reconstructs crystal structure, the photoisomerization of diarylethene is directly connected with the macroscopic deformation of the crystal^9, 10^. Beyond understanding the photoisomerization, predicting the population ratio of isomers is essential to identify the macroscopic deformation of diarylethene, and investigation of its deformation is necessary for photoactuator design performing complex operation.

Many groups have investigated the molecular design relevant to enhancing the material performance (e.g. thermal stability, quantum yield, and determining deformation type) in atomic-scale level^1, 3, 4, 6–16^, and studied mathematical modeling for analyzing photomechanical behavior of molecular crystals in continuum-scale level^17, 18^. In the atomic-scale part, currently reported isomerization dynamics^19–21^, such as Ehrenfest dynamics and surface hopping method, have shown relatively a little concern on reflecting external stimuli (light irradiation conditions, and temperature) despite of their significant influence on the photo-responsive performances. Continuum-scale studies^17, 18^ examined strain-stress-deformation relation on the basis of the theory of linear elasticity, and investigated the effect of various factors on photodeformation of single crystal, but the advancement should be followed in their chemical kinetic assumption on the isomerization ratio in that the photo-coupled reaction accompanied electronic structure change originating from the interaction energy between the light and electrons of the molecular system.

In this study, we propose quantum mechanical description on photoisomerization ratio (statistical model) reflecting external stimuli, and integrate the model with light penetration physics. As a result, penetration depth dependent isomerization progress is examined to predict photodeformation of diarylethene single crystal according to experimental deduction (proportional relationship between isomerization progress and photostrain)^4, 5^. As the mathematical base of the statistical model, the density matrix is composed of the photo-switching properties of the target molecule as well as external stimuli, and formulated as a state-space form to trace the population (existence probability of a certain molecular state) transition during isomerization. The photo-switching properties are obtained from first principle calculations (time-dependent density functional theory (TD-DFT), density functional theory (DFT), and complete active space self-consistent-field (CASSCF) calculations). Experimental comparisons^4, 22–26^ are given to confirm the reliability of our modeling.

## Results and Discussion

### Properties of the population transition path and photo-switching characteristics of diarylethene

**Figure 2:**
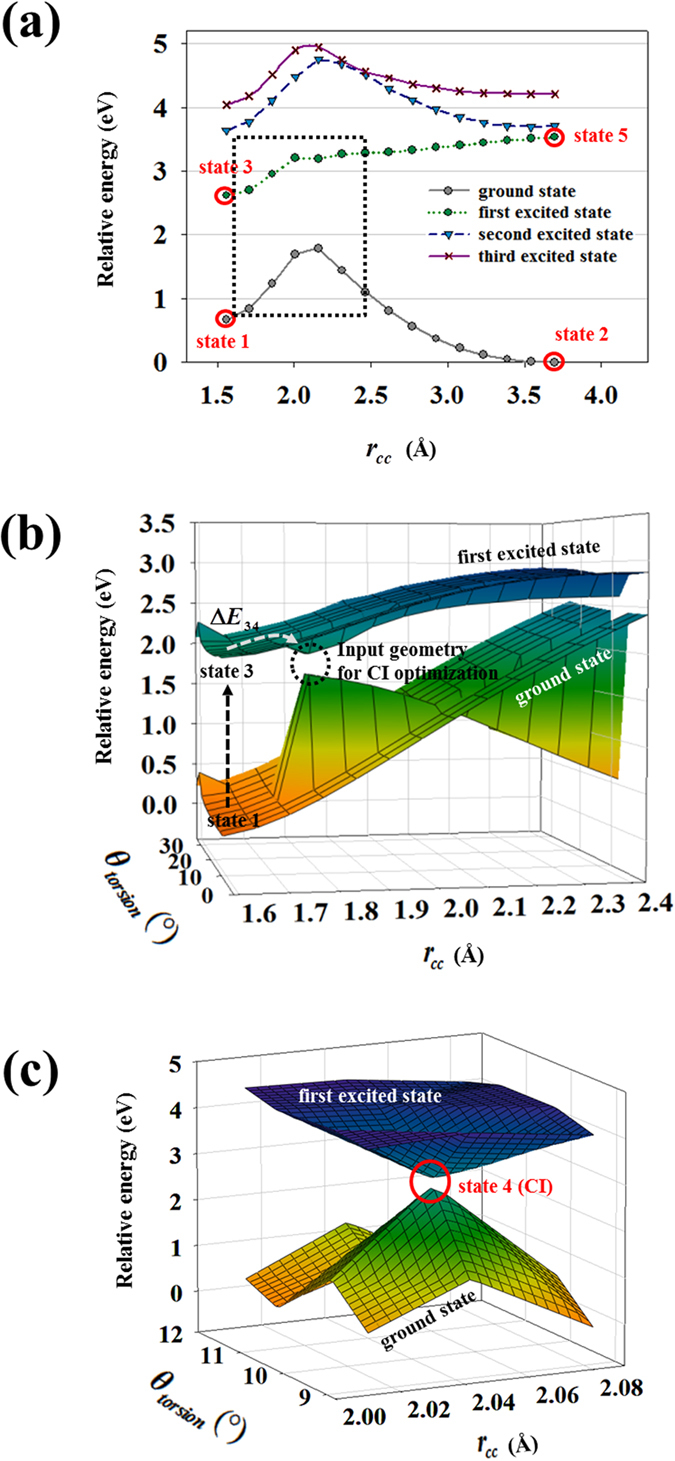
PESs of 1,2-bis(2-ethyl-5-phenyl-3-thienyl)perfluorocyclopentene. Highlighted red circles represent major molecular states. (**a**) Two-dimensional graph from geometry optimization and TD-DFT method is plotted for multiple energy states. Rectangular region with gray dotted line indicates the range of three-dimensional PES scanning. (**b**) Three dimensional PESs from geometry optimization and TD-DFT method containing the determined energy states from Fig. 2a. *ΔE*_*34*_ is the cycloreversion activation energy on first excited PES. (**c**) PESs around the CI by CASSCF calculations based on the location of the CI vicinal region from Fig. 2b.

In this section, the population transition path of diarylethene (Fig. 1c) during photoisomerization is investigated. The major molecular states involved in population transition are determined from the path, and their photo-switching properties are calculated to compose the density matrix equations. To identify the population transition path, the required data is divided into two parts: (1) determining the potential energy surfaces (PESs) governing the population transition, and (2) finding the location of the conical intersection (CI: state 4 in Fig. 1c) and exact activation energy of the chosen PESs. Two- and three-dimensional PES scans were performed to obtain the data for the effective computation. The two dimensional PES graph is plotted along the overall *r*_*cc*_ (the distance between the reacting carbon atoms as shown in Fig. 1b) range 1.558–3.697 Å (from closed ring to open ring, respectively), and with no constraint on *θ*_*torsion*_ (dihedral angle between the thiophene and perfluorocyclopentene as shown in Fig. 1b), as shown in Fig. 2a. Calculated energy barriers were too high for photoisomerization on most of the PESs, except the first excited energy surface as shown in Table 1. In the case of cyclization (ring-closing reaction in Fig. 1a), the ground state PES has a high energy barrier (1.127 eV), and the first excited PES has zero activation energy, corresponding to the thermal independence of the cyclization. For the cycloreversion (ring-opening reaction in Fig. 1a), the ground energy surface also has a high activation energy (1.789 eV), but the lowest energy barrier (0.586 eV) is found on the first excited energy surface.

**Table 1:** Energy barrier of each PES from the two-dimensional PES graph.

Energy barrier (eV)		
	Cyclization	Cycloreversion
Ground	1.789	1.127
First excited	0	0.586
Second excited	1.045	1.116
Third excited	0.739	0.904

Jacquemin *et al*. mentioned about the path along second excited PES of different type of derivative (bis(3-thienyl)perhydrocyclopentene) with ours. They predicate the path on that the open ring isomer can be excited to either first or second excited state based on similar oscillator strengths, 0.011 and 0.015 respectively^27^. In our case, oscillator strength of first excited state obtained by TDDFT calculation with B3LYP/6-31 G(d) level of theory is much larger than the value of second excited state (open ring isomer: first excited state-0.0365, second excited state-0.0064/closed ring isomer: first excited state-0.3719, second excited state-0.0029), and it demonstrates that the path containing second excited PES does not need to be concerned in our system.

Considering both the energy barriers and oscillator strengths, we conclude that the photo-switching reaction occurs through the first excited PES, instead of climbing up the energy barrier of the ground state. This also explains the thermal stability of the ground state of diarylethene, and necessity of light irradiation to stimulate the conversion between the open- and closed ring isomer.

Based on the above investigation, a three dimensional PES scan was performed for ground and first excited state energy surfaces in a restricted region (*r*_*cc*_ = 1.59–2.43 Å, *θ*_*torsion*_ = 8.8–34.18°) to reduce the computational cost (Fig. 2b). The activation energy *ΔE*_*34*_ of the cycloreversion on the PES of the first excited state is identified, and the value is 0.1073 eV (*r*_*cc*_ = 1.731 Å, *θ*_*torsion*_ = 16.358°). Tip point of the funnel (*r*_*cc*_ = 1.8 Å, *θ*_*torsion*_ = 20.08°, energy gap = 0.2328 eV) is used as an input geometry for the CI optimization by CASSCF calculations, because the avoided crossing with the small energy gap indicates that there exists a CI in the vicinity^28, 29^, and the starting point near the CI decreases the computational burden. Through the optimization of CI with CAS(6,6)/6-31 G(d), the exact CI (state 4, *r*_*cc*_ = 2.041 Å, *θ*_*torsion*_ = 9.681°) is located, and PESs (Fig. 2c, *r*_*cc*_ = 2.01–2.08 Å, *θ*_*torsion*_ = 8.8–11.62°) around the branching point obtained with CAS(10,10)/6-31 G(d) shows the reliability of the investigated CI (energy gap = 0.0255 eV).

In addition, qualitative similarities of PESs (Fig. 2) with other first principle studies^11, 19, 30–32^ strengthen its reliability, such as huge ground energy barrier, activation energy on first excited PES during ring-opening, barrierless first excited PES during ring-closing, and no-crossing between first and second excited PESs. These no-crossing PESs are also demonstrated in Figure S2 by equation of motion coupled-cluster singles and doubles (EOM-CCSD) method^33^ which involves double excitation operator.

The population transition path of the photoisomerization and five major molecular states (states 1–5 in Fig. 2) were determined from the results of the PES scan. The photo-switching properties of the obtained states (Table 2) were calculated to reflect the nature of target molecule in the density matrix equations (equations 1–3). Energy levels of the states (*E*_*1*_, *E*_*2*_, *E*_*3*_ and *E*_*5*_) are used to examine the excitation frequency from the ground to first excited state (equation 11). Vibrational relaxation rate constant *k*_*jn*_ determines its relaxation rate (state *j* → *n*) of the target molecule (equation 7). The electric transition dipole moment *d*_*mj*_ indicates polarization of spatially overlapped electrons between the ground and excited state, and composed the interaction energy *V*_*mj*_ between light and electrons (equation 8).

**Table 2:** Photo-switching properties of diarylethene (each variable listed in equations 4–11).

Energy level (eV)			
***E***_**1**_	***E***_**2**_	***E***_**3**_	***E***_**5**_
0.662	0	2.619	3.537
**Vibrational frequency (cm** ^**−1**^ **)**			
***k***_**34**_	***k***_**54**_	***k***_**41**_	***k***_**42**_
221.14805	55.17	271.96	271.96
**Electric transition dipolemoment (a.u.)**			
**|** ***d***_**13**_ **|**		**|** ***d***_**25**_ **|**	
2.784		0.649	
**Energy barrier (eV)**			
Δ*E*_34_ = 0.1073			

Using equations 4–11 with the obtained photo-switching properties, we derived the component terms (Table S3) of density matrix equations relevant to spontaneous decay and vibrational relaxation. *f*_*boltzmann*_ indicates the Boltzmann factor in equation 7.

### Population evolution of molecular states of diarylethene

In the following section, the time-evolutional population profile of each state is calculated based on the statistical photoisomerization model, and validated by comparing with the transient-absorption experiment^22–26^.

In this study, the statistical photoisomerization model (equations 1–3) enables tracing the population of state *j* (*ρ*_*jj*_) for *j* = 1–5 (see Fig. 1c) during the isomerization, and is composed of both the photo-switching properties listed in Table 2 and the prescribed external stimulus factors (*n*_*ω*_: the number of photons per second, *θ*: included angle between polarized light and electric transition dipole moment, *T*: temperature in equations 4–11). Assuming resonance excitation, the wavelengths of light are set to 350.58 nm for UV excitation, and 633.38 nm for visible light excitation, which are derived from the energy difference between the ground and first excited state of the each isomer. The light intensity (*n*_*ω*_: *n*_*uv*_ for UV light and *n*_*vis*_ for vis light) applied in the model is 2.0*10^26^ photons/s (UV: 20.517 MW/cm^2^, vis: 3.4794 MW/cm^2^). Its high intensity accelerates the population transition, and enables the simulation with reasonable computational cost^34, 35^. The transition does not involve multi-photon excitation, because we restricted its path to that of the single-photon excitation mode (Fig. 1c). Other external stimulus factors are (*θ*: *θ*_*UV*_ for UV light and *θ*_*vis*_ for vis light) = 0° and *T = *300 K.

**Figure 3:**
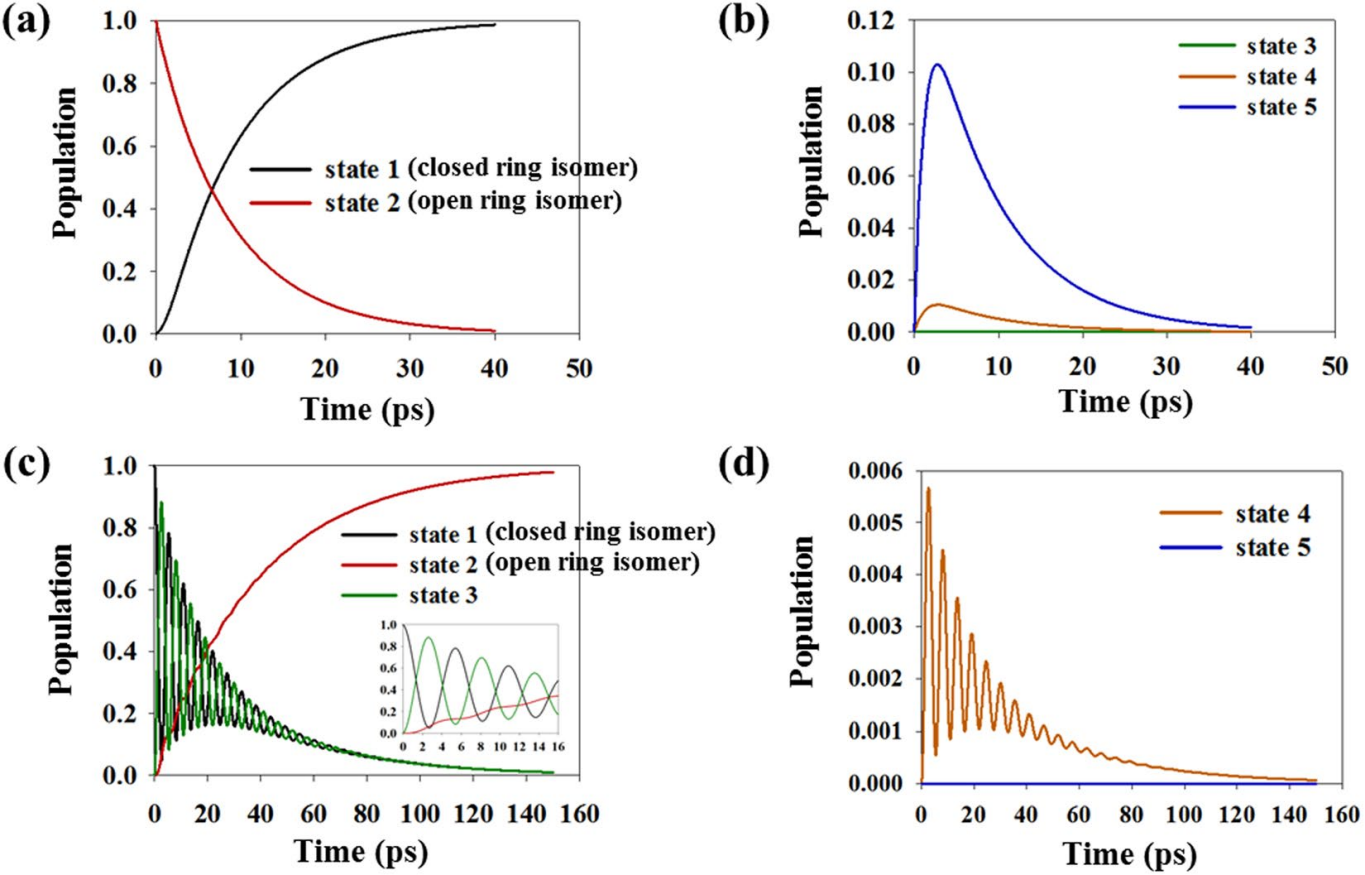
(**a**,**b**) Population of each molecular state for cyclization (*n*_*uv*_ = 2.0*10^26^ photons/s, and *θ*_*UV*_  =  0). (**c**,**d**) Population of each molecular state for cycloreversion (*n*_*vis*_ = 2.0*10^26^ photons/s, *θ*_*vis*_ = 0, and T = 300 K). Early time data (~16 ps) is shown in inset (*n*_*uv*_ and *n*_*vis*_ are the number of photons per second. *θ*_*UV*_ and *θ*_*vis*_ indicate light polarization condition).

Through statistical isomerization model, both the cyclization and cycloreversion processes are represented as the population term in Figure 3. Figure 3a and b are obtained through the cyclization simulation, with *n*_*uv*_ = 2.0*10^26^ photons/s and *θ*_*UV*_ = 0. When irradiated with UV photons, the population is excited from state 2 to state 5. The population then either decays to state 2 or relaxes vibrationally to state 1 via state 4. As a result of the transition, the population of state 2 completely travels to the state 1. The small amount of the population in the intermediate states (states 4 and 5) indicate that most of the population does not lag but passes through them (see Figs 1c, 3a and b).

In the case of cycloreversion (external stimuli: *n*_*vis*_ = 2.0*10^26^ photons/s, *θ*_*vis*_ = 0°, Temperature = 300 K), population data of each state is plotted in Fig. 3c and d. Irradiating with visible photons stimulates the excitation (state 1 → 3), and is followed by either spontaneous decay (state 3 → 1) or vibrational relaxation (state 3 to 2 through state 4). The longer reaction time is identified from a comparison with the cyclization results, and an oscillation profile which is similar to the harmonic oscillation of the two-state excitation model^36, 37^ is also found. The population of state 4 has a small value similar to the results shown in Fig. 3b, but also shows an oscillation profile caused by the influence of state 3 (see Figs 1c, 3c and d).

The reliability of our statistical photoisomerization model is validated by comparison with experimental results (transient-absorption spectroscopy)^22–26^. To identify the pulsed laser irradiation conditions from the experiment, the starting point of the population transition is set to the excited state of initial isomer in the model. The reactants and products of the isomerization are trivial as a result of the simulation, so the rate of the reaction should be compared to verify our model.

**Figure 4:**
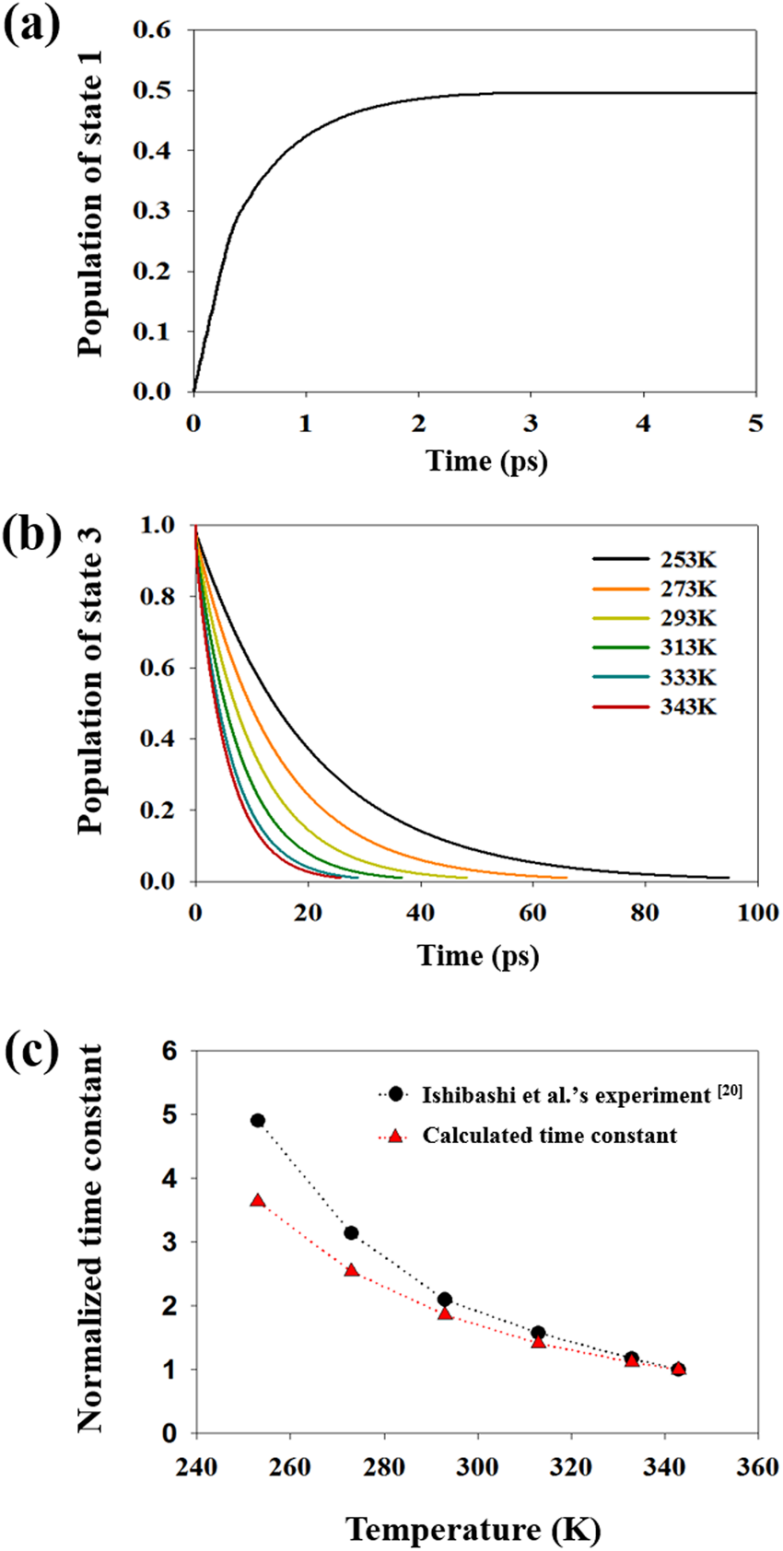
(**a**) Population of the ground state of the closed ring isomer under UV pulse laser irradiation (cyclization). (**b**) Population of the first excited state of the closed ring isomer according to the temperature under vis pulse laser irradiation (cycloreversion). (**c**) Temperature dependency of time constants of calculated population under vis pulse laser irradiation. The constants of our model and Ishibashi *et al*.^23^ are normalized by each value at 343 K.

Thus, the time constant, which is the reciprocal of the reaction rate, is relevant for the comparison. The time constants of the population evolutions of state 1 and 3 (see Fig. 4) were obtained for the cyclization and cycloreversion processes, respectively. These states were chosen because the experimental value indicates the time constant of transient absorbance, which consists of the molecular concentration of a certain state (state 1 for the cyclization, state 3 for the cycloreversion)^38^. For the cyclization, the time constant was attained from the population profile of state 1 (Fig. 4a) irradiated by a UV laser pulse. The time constant (0.59 ps) of the theoretical model is within in a few picoseconds of experimental data^22, 25, 26^ for various diarylethene derivatives, and the model simulated reliable population transition processes for the cyclization.

In the case of cycloreversion under pulsed visible laser irradiation, the time constants (Table S4) are calculated from the population profile of state 3 (Fig. 4b) according to the temperature (253–343 K). To examine the temperature dependency of our data, time constants are normalized (Fig. 4c) by each value at 343 K because of difference in derivatives (ours: 1,2-bis(2-ethyl-5-phenyl-3-thienyl)perfluorocyclopentene, Ishibashi *et al*.^23^: 1,2-bis(2-mehtyl-3-benzothienyl)perfluoro cyclopentene). The constants from the theoretical model are inversely proportional to the temperature, and their dependency profile according to the temperature is very similar to the experiment^23^. Its similarity is verified by non-radiative transition simulation of both derivatives in Figures S3 and S4. The magnitude of 10.565 ps at 293 K is especially close to the experimental data^24^ of 10 ps at 295.15 ± 2 K. This indicates that our statistical photoisomerization model reliably reproduces the physics of the population transition for cycloreversion as well as for the cyclization.

The validated statistical isomerization model is parametrized (equations 12–14) for computational convenience of light penetration coupling simulation in photodeformation section. In addition, we provided crucial findings (primary stimuli and molecular design parameter for increasing the reaction rate, and external condition for enhancing fluorescence performenace of 1,2-bis(2-mehtyl-3-benzothienyl)perfluorocyclopentene) which are solely obtained by the statistical model in Figures S5–S8.

### Predicting photodeformation of diarylethene single crystal

When the material is irradiated by light, the intensity of light has decaying profile along its penetrating direction (Beer’s law), and it causes penetration depth-dependent isomerization progress. As a result, its isomerization profile according to the penetration depth determines specific type of photodeformation such as in-plane or bending deformation.

**Figure 5:**
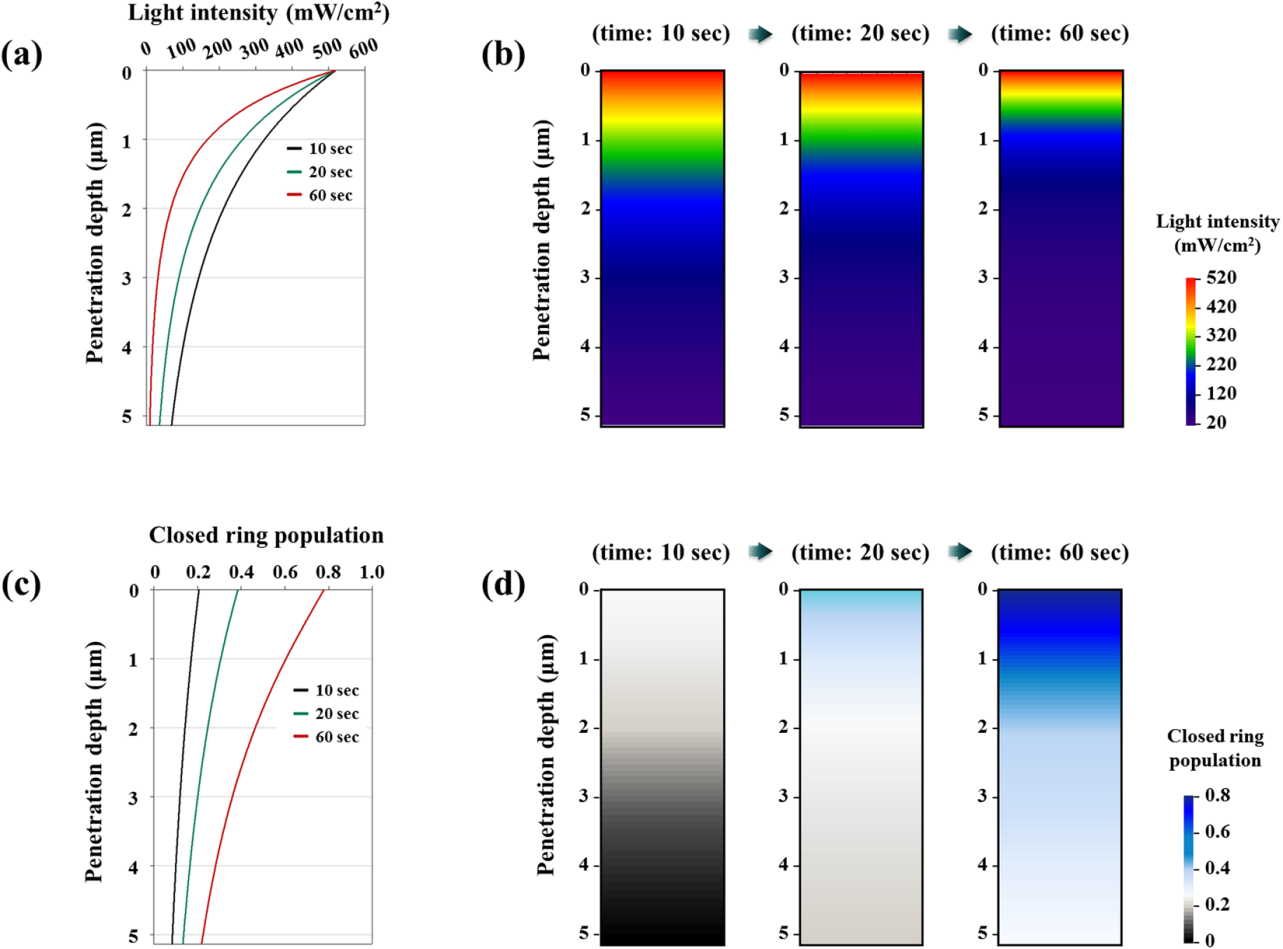
(**a**,**b**) Penetration depth-dependent light intensity profile. (**c**,**d**) Penetration depth-dependent population of ground closed ring isomer. (Ground open ring isomer occupies the rest of closed ring population).

In this section, the light penetration-coupled statistical photoisomerization model (equations 12–16, Figure S9) was applied to investigate population of both isomers and light intensity profile according to the penetration depth (Fig. 5). Prescribed stimuli were *n*_*uv.0*_ (5.069*10^18^ photons/s ~520 mW/cm^2^ at 350.58 nm) and *θ*_*UV*_ (non-polarized light). The thickness was set to 5.135 μm.

In the initial stage of light penetration, its intensity decreases as the distance from the surface becomes deeper as shown in Fig. 5a and b according to the Beer’s law (equation 15). Strong light intensity near the surface promotes cyclization, and closed ring isomers are congested at the upper part of the material (Fig. 5c and d). As irradiation time elapsed, over-crowded closed ring isomers near the surface absorb large amount of UV photons (absorbance at 350.58 nm: closed ring isomer ≫ open ring isomer), and it delays cyclization progress at the lower part of material. Consequently, most of open ring isomers are converted into closed ring isomers (~72.68% conversion at 60 seconds) within 0.57 μm distance from the surface, and poor conversion ratio (~28.21% conversion at 60 seconds) is found at the bottom part (3–5.135 μm distance from the surface).

According to experimental studies^4, 5^, photostrain is directly affected by the amount of isomerization progress. Based on this deduction, the penetration depth-dependent population (Fig. 5c and d) nicely explains bending deformation of thick sample (diarylethene single crystal with approximate 5 μm thickness)^4^ where large strain is applied near the surface but small strain yields on the lower part. It also clearly describes in-plane deformation of thin sample (diarylethene single crystal having thickness less than 1 μm)^4^ in that relatively constant isomerization progress (70–80% conversion at 60 seconds) is estimated at the upper part (0–0.57 μm distance from the surface) in Fig. 5c and d.

To derive proportional constant (*c* in equation 17 and Figure S10) which is essential for the prediction of photodeformation, we referred experimental data (corner angle *θ*_*corner*_ of lozenge-shaped crystal in Figure S10 is 82° under UV irradiation of 520 mW/cm^2^ at 60 seconds)^4^, and also examined cyclization conversion ratio ((*ρ*_*o*→*c*_)_*avg*._: 0.7268 at 60 seconds from Fig. 5c). As a result, the proportional constant (*c*: 0.8043*β) was obtained, and used to predict shear deformation under UV irradiation.

**Figure 6:**
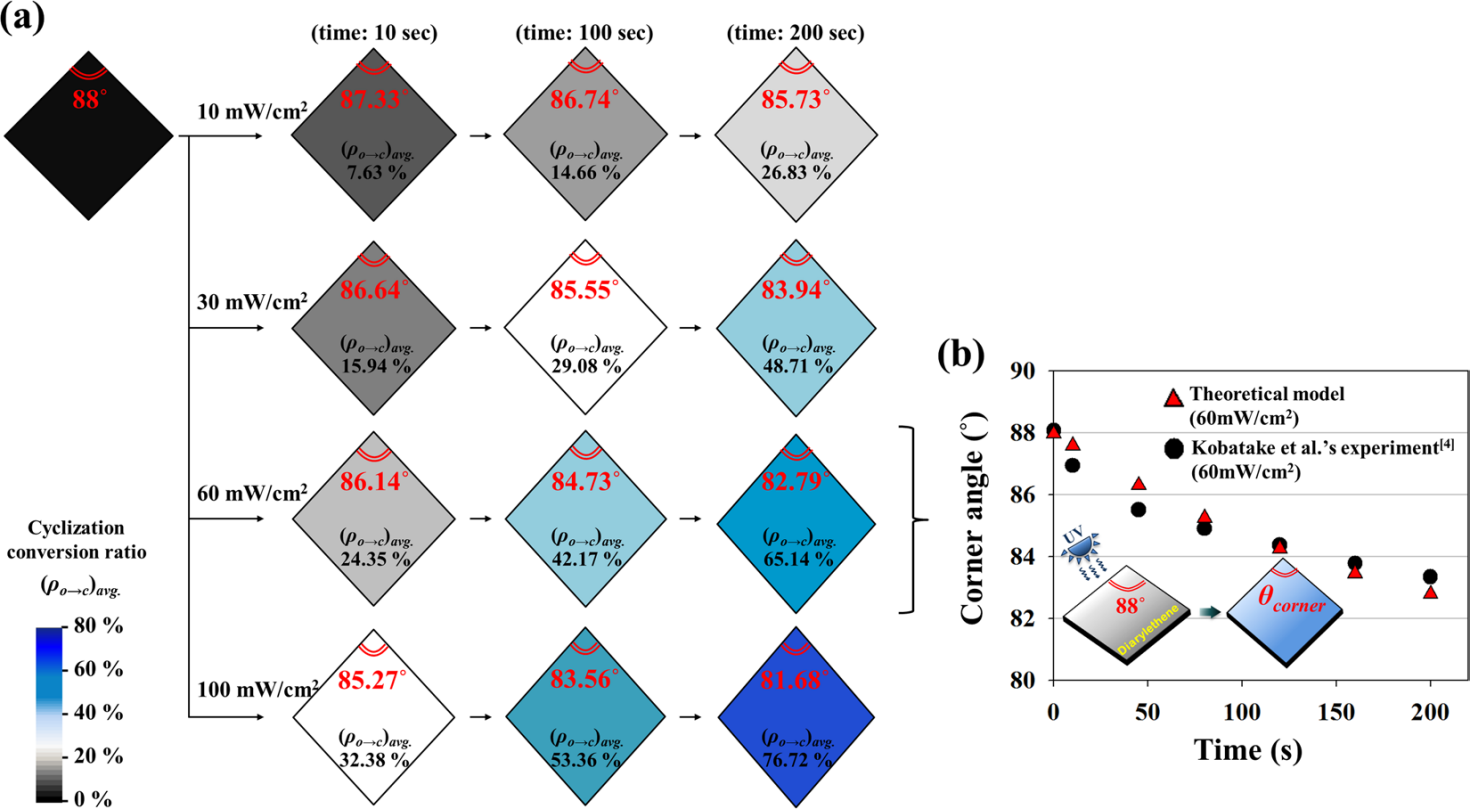
(**a**) Predicted in-plane shear deformation induced by UV irradiation. Corner angle and cyclization conversion ratio of lozenge-shaped single crystal are indicated in insets. (sample: 1,2-bis(2-ethyl-5-phenyl-3-thienyl)perfluorocyclopenetene single crystal, thickness: 0.57 μm, light wavelength: 350.58 nm, light intensity: 10–100 mW/cm^2^) (**b**) Experimental comparison (red triangular point: Kobatake *et al*.’s report^4^) with our model at 60 mW/cm^2^ of UV irradiation.

The light penetration-coupled statistical photoisomerization model was employed to examine the cyclization conversion ratio, (*ρ*_*o*→*c*_)_*avg*._, with 9.75*10^16^–9.75*10^17^ photons/s (~10–100 mW/cm^2^ at 350.58 nm) of *n*_*uv.0*_. Finally, corner angle (*θ*_*corner*_) of our system was investigated based on 4 known variables ($\overline{{{\rm{A}}}_{{\rm{0}}}{{\rm{B}}}_{{\rm{0}}}}$, $\overline{{{\rm{A}}}_{{\rm{1}}}{{\rm{C}}}_{{\rm{1}}}}$, *c* and (*ρ*_*o*→*c*_)_*avg*._ in equation 17 and Figure S10) in Fig. 6a. As the single crystal is irradiated by the strong intensity of light, it showed rapid shear deformation corresponding to fast cyclization conversion. Especially, computational value at 60 mW/cm^2^ of UV irradiation has a good agreement with experimental data^4^, both qualitatively and quantitatively (Fig. 6b). In addition, we also constructed polarization effect-embedded theoretical model (Figure S11) which was developed from the presented model (non-polarized light source), and experimental comparison was given for validation of its reliability. These theoretical predictions according to various light irradiation conditions would provide guideline for photoactuator design conducting complicated operation.

In summary, mathematical model, density matrix formulation, combined with first principle calculations (geometry optimization, TDDFT, CASSCF, and frequency calculation in Fig. 2 and Table 2) was suggested to investigate photoisomerization using probabilistic representation, and it enabled the conditions of light irradiation and temperature to be investigated. Its quantum mechanical description brought advancement to the currently reported kinetic assumption on photoisomerization ratio^17, 18^, and quantified population of each isomer under various external stimuli solely through the theoretical approach (Fig. 3, S5 and S6). We also integrated the statistical photoisomerization model with light penetration physics which realized the feedback relation between the light penetration and the isomerization ratio, and it well reproduced the light penetration intensity profile and penetration depth-dependent isomerization progress (Fig. 5). They were used to predict accurate computational value of in-plane shear deformation (corner angle of lozenge-shaped single crystal during cyclization in Fig. 6) according to the experimental report^4^. Polarization effect-reflected isomerization ratio was also investigated (Figure S11), and its reliability was validated by experimental comparison.

In addition, the statistical model itself provided molecular design parameters determining the isomerization rate and external stimuli (generating metastable closed ring state) enhancing the fluorescence performance (Figures S7 and S8). Its reliability was validated by comparing time constant of the computational model with that of the transient absorption spectroscopy^22–26^ (Fig. 4 and Table S4).

We believe that establishment of the light penetration-coupled statistical photoisomerization model giving the ratios for the populations of the quantitative isomerization progression will contribute the development of advanced photoactuator, and help diarylethene be useful as an industrial application from the current experimental stage.

## Methods

### First principle calculation

In all first principle calculations (DFT, TD-DFT, and CASSCF), the Gaussian 09 package^39^ was used for unimolecular modeling, and solvent interaction was not considered during the calculations. X-ray crystallography data obtained by Kobatake *et al*.^6^ was used for the starting geometries of both open- and closed-ring 1,2-bis(2-ethyl-5-phenyl-3-thienyl)perfluorocyclopentene used in the calculations.

The main goal of the first principle calculations in this paper is finding the population transition path during the isomerization, and determining the photo-switching properties of diarylethene from the path. As population transitions occur along the path via major molecular states (ground and excited states for both open- and closed ring isomers, and conical intersections), we conducted a PES scan to identify the population transition path; this data is essential for calculating the properties of the states. The PES scan was performed for the ground and excited states of the molecule along the reaction coordinate. The reaction coordinate (Fig. 1b) indicates both *r*_*cc*_ and *θ*_*torsion*_, and the former is generally considered a crucial factor for the geometry change of the ring structure.

During scanning PES for the ground state, geometry optimizations were conducted for every scanning sample at the B3LYP^40–42^/6-31 G(d)^43^ level of theory with the target reaction coordinates fixed as described above. In the case of the PES scans for the excited states, TD-DFT calculations were performed for every optimized molecular geometry in the ground state, with the assumption that vertical excitation occurs from the ground state to reduce the computational cost. The TD-DFT calculations were performed with the B3LYP^40–42^/6-31 G(d)^43^ functional/basis set to estimate the singlet excitation states for the molecule.

Based on the PES obtained by DFT and TD-DFT methods, CASSCF calculations were conducted to optimize the exact CI and scan the PES with the assumption of vertical excitation around the branching point, because the method provides the precise PESs especially in the region of vicinal CI^11^. CAS(6,6)/6-31 G^44, 45^ was used during the optimization of the CI to reduce the high computational cost, and CAS(10,10)/6-31 G(d) was employed for the PES scanning around the branching point. Active space was determined in reference to Woodward-Hoffmann rules^46^ that the photoisomerization is a electrocyclic reaction with (4n + 2) π-orbitals, and dependency of the system on the number of active spaces and orbital basis was shown in Table S2.

The UV/vis absorption spectra were calculated for both open- and closed-ring isomers of diarylethene and compared with experimental data^6^ to validate the reliability of the orbital basis set, as shown in Figure S1. Oscillator strength is a calculated quantity that corresponds with absorbance and describes the tendency of electrons to show radiative transitions by absorption of light^11^. By comparing the longest wavelength at the peaks of the spectra of the calculation with experiment, we assure that our calculation (B3LYP/6-31G(d)) results are well-matched with experimental results^6^ among the various functional/orbital basis in Table S1. The calculated values of 285.06 nm for the open ring structure and 633.38 nm for the closed ring are in agreement with the experimental values^6^ of 286 nm for the open ring structure and 600 nm for the closed ring structure, showing that the excitation of the diarylethene molecule is well-predicted.

After the PES scanning, we identified the population transition path, and defined the major molecular states along the path. Photo-switching properties (e.g. energy levels, electric transition dipole moments, vibrational relaxation rate constants, and energy barriers) of each state were calculated at the B3LYP^40–42^/6–31 G(d)^43^ level of theory to construct the density matrix equations.

The ground state energy levels were obtained from the geometry optimization. The TD-DFT calculations investigate the first excited energy levels and the electric transition dipole moment. The calculation method for vibrational relaxation rate constants depends on the existence of the energy barrier along the transition path. In the case of spontaneous relaxation during the transition (state 5 → 4, state 4 → 1, state 4 → 2), imaginary frequency showing ring opening-closing mode is examined to derive the rate constant (*k*_*41*_, *k*_*42*_ and *k*_*54*_ in Table 2)^47^. For the transition path (state 3 → 4) including the energy barrier, normal mode frequencies was investigated for obtaining the rate constant (*k*_*34*_ in Table 2) based on transition state theory^48^. The value of energy barrier is examined by PES scanning.

### Statistical photoisomerization modeling

The statistical photoisomerization model is based on density matrix formalism composed of both photo-switching properties and relevant terms for external stimuli. A density matrix is a probabilistic representation indicating a statistical ensemble of multiple molecular states, and it was originally used to describe the population transitions among individual states in atomic or molecular systems interacting with electromagnetic waves^36, 37, 49^. Herein, we propose combining the statistical photoisomerization model with the density matrix formalism to simulate the population transitions stimulated by continuous light irradiation, which involve major molecular states. The expression of the density matrix equations in the model refers to radiative transition theory of Krainov *et al*.^50^.

In the first principle calculations, the major states are examined and defined, as shown in Figs 1c and 2. The states 1 and 2 are ground states of the closed- and open-ring isomers, and states 3 and 5 are the first excited states of closed- and open-ring isomers. State 4 is the CI indicating the point where two energy surfaces are degenerate, and a radiationless (non-adiabatic) transition occurs from the excited state to the ground state surface.

The population transition consists of three processes: excitation, spontaneous decay, and vibrational relaxation, and its path varies with the type of isomerization (Fig. 1c). For example, considering the cyclization (ring closing isomerization), UV light irradiation stimulates excitation (state 2 → 5) then vibrational relaxation (state 5 → 4 → 1, or state 5 → 4 → 2), or spontaneous decay (state 5 → 2) subsequently occurs. As a result, the entire population transfers to state 1 with continuous light irradiation. In case of the cycloreversion (ring opening isomerization), visible light promotes the excitation (state 1 → 3), then vibrational relaxation (state 3 → 4 → 2 or state 3 → 4 → 1) or spontaneous decay (state 3 → 1) follow, and finally the population transfers completely to state 2 with continuous irradiation of light.

In order to interpret the population transition physics, the statistical isomerization model of each state with the density matrix formalism is constructed as shown in equations 1 and 2:

1 $$\frac{d{\rho }_{jj}}{dt}+({{\Gamma }}_{jj}+{{{\rm K}}}_{jj}){\rho }_{jj}-\sum _{p}\,[({\gamma }_{pj}+{\kappa }_{pj}){\rho }_{pp}]=\frac{i}{\hslash }\sum _{m}\,[({V}_{mj}{\rho }_{jm}-{V}_{jm}{\rho }_{mj})]$$

2 $$\frac{d{\rho }_{jl}}{dt}+({{\Gamma }}_{jl}+{{{\rm K}}}_{jl}+i{{\rm{\omega }}}_{jl}){\rho }_{jl}=\frac{i}{\hslash }\sum _{m}\,[({V}_{ml}{\rho }_{jm}-{V}_{jm}{\rho }_{ml})]$$

which indicate the first derivatives of population in terms of *ρ*_*jj*_ and *ρ*_*jl*_. The population of each state (*j* = 1–5) is indicated as *ρ*_*jj*_, and *ρ*_*jl*_ represents phase change term of the excitation (*jl* = 13, 31, 25, 52). Using the linearized equations 1 and 2, the state-space matrix form is established to track the population evolution of each state, as shown in equation 3.

3 $$[\begin{array}{c}{\dot{\rho }}_{jj}\\ {\dot{\rho }}_{jl}\end{array}]=[M][\begin{array}{c}{\rho }_{jj}\\ {\rho }_{jl}\end{array}]$$

Among the three transition processes, excitation is represented as the right hand side of equations 1 and 2, and spontaneous decay is reflected by the **Γ** term, and vibrational relaxation is represented by the **Κ** term. The spontaneous decay rate from state *j* to state *m* is represented by the term *γ*_*jm*_, which is a composition term of **Γ** (equations 4 and 6). The relaxation rate from state *j* to state *n* is represented by *κ*_*jn*_, which is a component of **Κ** (equations 5 and 7), and consists of *k*_*jn*_ (vibrational relaxation rate constant for state *j* → *n*) with the Boltzmann factor (*k*_*B*_ = Boltzmann constant). The energy barrier from state *j* to state *n* is shown by *ΔE*_*jn*_, and *T* is the temperature in degrees Kelvin.

4 $${\Gamma }_{jl}=\sum _{m}({\gamma }_{jm}+{\gamma }_{lm})$$

5 $${{\rm{{\rm K}}}}_{jl}=\sum _{n}({\kappa }_{jn}+{\kappa }_{ln})$$

6 $${\gamma }_{jm}=\frac{4{{\omega }^{3}}_{jm}}{3\hslash {c}^{3}}{|{d}_{jm}|}^{2}$$

7 $${\kappa }_{jn}=\frac{{k}_{jn}}{2}{e}^{\frac{-{\rm{\Delta }}{E}_{jn}}{{k}_{B}T}}$$

8 $${V}_{mj}=-\varepsilon \cdot {d}_{mj}=-|({\varepsilon }_{0}\,\cos ({\omega }_{mj}t)){d}_{mj}|\cos \,\theta $$

9 $${\varepsilon }_{0}=\sqrt{\frac{4\hslash {{\omega }^{3}}_{jl}{n}_{\omega }}{\pi {c}^{3}}}$$

10 $${\rho }_{jl}={{\Psi }}_{j}\cdot {{\Psi }}_{l}^{\ast }={a}_{j}(t)\cdot {a}_{l}^{\ast }(t){e}^{i{\omega }_{lj}t}$$

11 $${{\rm{\omega }}}_{jl}=\frac{{E}_{j}-{E}_{l}}{\hslash }$$

In the representation of the excitation, *V*_*mj*_ is a key component for the driving source of the excitation. This is an interaction energy between the light and electrons as shown in equations 8 and 9, where *ε* is the electric field strength of the light, *ε*_*0*_ is the magnitude of electric field strength of the light, and *θ* is the included angle between the polarized light and the electric transition dipole moment. The light intensity and light polarization are represented by *ε*_*0*_ and *θ* respectively, and impose the light irradiation condition in the *V*_*mj*_ term. The number of photons arriving at the molecule per second (*n*_*ω*_) can be interpreted using *n*_*uv*_ for the UV light or *n*_*vis*_ for the visible light in the model.

Other variables (equations 4–11) in the statistical isomerization model are: *Ψ*_*j*_ is the time-dependent wave function of electrons in state *j*, *E*_*j*_ is the energy level of state *j*, *d*_*mj*_ is the electric transition dipole moment for the excitation, *mj* = 13 or 25, and *ω*_*jl*_ is the excitation frequency from state *j* to state *l*. In this calculation, *ω*_*jl*_ has the same value with the light frequency *ω*_*mj*_, assuming resonance conditions for the convenience of the calculations. The constants *ћ* and *c* are the reduced Planck’s constant and speed of light, respectively. From the overall density matrix equations, the photo-switching properties *E*_*j*_, *ΔE*_*jn*_, *d*_*mj*_, and *k*_*jn*_ from the first principle calculations, combined with the prescribed external stimulus factors *n*_*ω*_, *θ*, and *T*, compose the statistical isomerization model. Thus, photoisomerization reflecting thermal/light stimuli can be analyzed with the parametric study of *n*_*ω*_, *θ*, and *T*.

### Light penetration coupling

We parametrized statistical photoisomerization model (cylization reaction, e.g. Fig. 3a and b) for computational convenience as shown in equations 12–14, and non-polarized light condition was applied. Population of ground open- and closed ring state are indicated as *ρ*_*open*_ (~*ρ*_*22*_ in equation 1) and *ρ*_*closed*_ (~*ρ*_*11*_ in equation 1), respectively. *n*_*uv*_ represents the number of UV photons (at 350.58 nm) per second.

12 $${\rho }_{open}={e}^{-[({10}^{\alpha }-1)\ast t]}$$

13 $${\rho }_{closed}=1-{\rho }_{open}$$

14 $$\alpha =10.95\ast {e}^{-{(\frac{\mathrm{log}({n}_{uv}+1)-28.3}{3.65})}^{2}}$$

According to experimental studies^4, 6^, each isomer of diarylethene has different absorbance at UV region (350.58 nm), and it lets light penetration profile be influenced by the amount of population of each isomer (*ρ*_*open*_ and *ρ*_*closed*_). Thus, light intensity (*n*_*uv*_) with respect to the penetration depth (z) is represented as shown in equation 15 according to the Beer’s law, and its total absorption coefficient (*k*_*uv*_) consists of population of each isomer (*ρ*_*open*_ and *ρ*_*closed*_) in equation 16. Absorption coefficient of each isomer at 350.58 nm is referred from experimental report^4^, and their values are *k*_*uv.open*_ (1.987*10^5^ m^−1^) and *k*_*uv.closed*_ (1.542*10^6^ m^−1^).

15 $$\frac{d{n}_{uv}}{dz}=-{k}_{uv}{n}_{uv}$$

16 $${k}_{uv}={k}_{uv.open}{\rho }_{open}+{k}_{uv.closed}{\rho }_{closed}$$

As a result, light penetration physics (equations 15 and 16) is integrated with parametrized statistical photoisomerization model (equations 12–14), and penetration depth-dependent isomerization progress is derived as a population term through iteration algorithm in Figure S9. In each single iteration step, the total absorption coefficient (*k*_*uv*_) is updated by substituting current population of each isomer to realize the feedback relationship between the light penetration and the ratio of the isomers.

Photodeformation of our system (1,2-bis(2-ethyl-5-phenyl-3-thienyl)perfluorocyclopenetene single crystal with 0.57 μm thickness) is in-plane shear deformation as shown in Figure S10, and diagonal contracts along $\overline{{{\rm{A}}}_{{\rm{1}}}{{\rm{B}}}_{{\rm{1}}}}$ direction^4^.

17 $$\begin{array}{rcl}\overline{{{\rm{A}}}_{1}{{\rm{B}}}_{1}} & = & \overline{{{\rm{A}}}_{0}{{\rm{B}}}_{0}}-c\ast {({\rho }_{o\to c})}_{avg.}\\  & = & \overline{{{\rm{A}}}_{1}{{\rm{C}}}_{1}}\,\sin \,{\theta }_{corner}\end{array}$$

Experimental group inferred that the photostrain induced by UV light would be proportional to cyclization progress^4, 5^, and the length of contracted diagonal ($\overline{{{\rm{A}}}_{{\rm{1}}}{{\rm{B}}}_{{\rm{1}}}}$) can be mathematically represented as equation 17 on the assumption of constant length of single crystal outline ($\overline{{{\rm{A}}}_{{\rm{1}}}{{\rm{C}}}_{{\rm{1}}}}$). The cyclization conversion ratio, (*ρ*_*o*→*c*_)_*avg*._, is estimated by averaging closed ring population within 0.57 μm thickness, and *c* indicates proportional constant between the contracted length and the amount of cyclization progress. *θ*_*corner*_ is corner angle of lozenge-shaped single crystal in Figure S10. To derive proportional constant (*c*), we referred experimental data^4^ with computed cyclization conversion ratio in Results and Discussion section (Predicting photodeformation of diarylethene single crystal).

## Electronic supplementary material

Supplementary Information
