# Supplementary material for: Light penetration-coupled photoisomerization modeling for photodeformation of diarylethene single crystal: upscaling isomerization to macroscopic deformation
Source: Sci Rep. 2017 Apr 19;7:967. doi: 10.1038/s41598-017-00910-5 (PMC5430494; doi:10.1038/s41598-017-00910-5)
Supplement: Supplementary file 1 — Supplementary Information [file 41598_2017_910_MOESM1_ESM.docx]

**Supplementary Information on**

**“Light penetration-coupled photoisomerization modeling for photodeformation of diarylethene single crystal: upscaling isomerization to macroscopic deformation”**

Muyoung Kim^1^, Jung-Hoon Yun^1^, and Maenghyo Cho^1,*^

^1^ Divison of Multiscale Mechanical Design, School of Mechanical and Aerospace Engineering,

Seoul National University, Seoul, Republic of Korea

**1. Details of reliability test of orbital basis set**

Various functional/orbital basis sets (split valence, diffuse, and polarized basis) were investigated to determine optimal calculation condition. Polarizable continuum model (PCM) with n-Hexane solvent was also employed for identical condition with the experiment^1^.

By comparing the longest wavelength at the peaks of the spectra with the experimental value^1^ (open ring isomer: 286 nm, closed ring isomer: 600 nm), the reliability of the orbital basis can be justified, and it is indicated in the Figure S1 and Table S1. Considering the gap between the calculated and experimental wavelengths, 6-31G(d) was the most suitable to reproduce the excitation of our system, and its reliability can be assured by comparing with Staykov *et al.*’s works^2,3^ which indicate either similar or larger magnitude of the gap than ours. Therefore, all first principle calculations employed B3LYP/6-31G(d) functional/basis set.


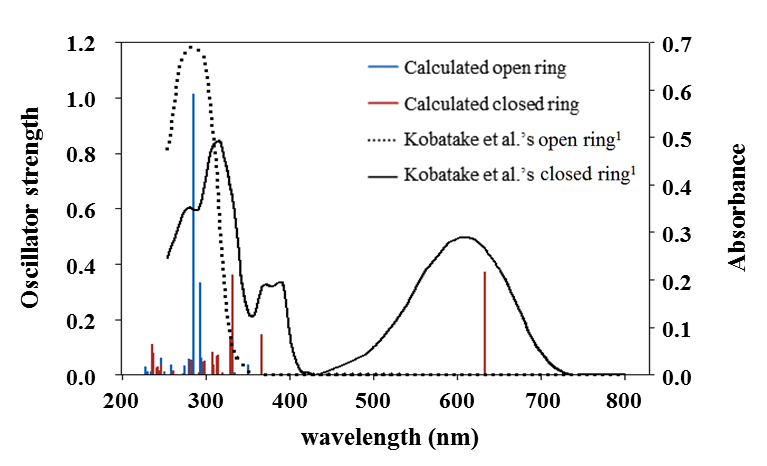


**Figure S1.** Calculated UV/vis absorption spectra of open- (blue bar) and closed ring isomer (red bar) are plotted with experimental spectra of Kobatake et. al.^1^ (open ring: dotted line, closed ring: solid line). Calculated data represent oscillator strength by B3LYP/6-31G(d), and experimental value indicates absorbance according to the wavelength.

**(a)**

| Level of theory  (open ring isomer) | PBE/  6-31G(d) | PBE/  6-31+G(d) | B3LYP/  6-31G(d) | B3LYP/  6-31+G(d) | CAM-B3LYP/  6-31G(d) | CAM-B3LYP/  6-31+G(d) |
| --- | --- | --- | --- | --- | --- | --- |
| **Wavelength (nm)** | 310.75 | 318.26 | 285.06 | 291.6 | 266.77 | 274.33 |
| **Level of theory**  **(closed ring isomer)** | PBE/  6-31G(d) | PBE/  6-31+G(d) | B3LYP/  6-31G(d) | B3LYP/  6-31+G(d) | CAM-B3LYP/  6-31G(d) | CAM-B3LYP/  6-31+G(d) |
| **Wavelength (nm)** | 723.02 | 744.04 | 633.38 | 651.43 | 558.71 | 573.77 |

**(b)**

| Orbital basis set | Wavelength (nm) | Orbital basis set | Wavelength (nm) |
| --- | --- | --- | --- |
| 6-31G(d) | 285.06 | 6-31G(2d,p) | 286.68 |
| 6-31+G(d) | 291.6 | 6-31+G(2d,p) | 293.15 |
| 6-31++G(d) | 291.62 | 6-31++G(2d,p) | 293.16 |
| 6-311G(d) | 288.93 | 6-311G(2d,p) | 289.94 |
| 6-311+G(d) | 292.65 | 6-311+G(2d,p) | 293.78 |
| 6-311++G(d) | 292.65 | 6-311++G(2d,p) | 293.8 |
| PCM/6-31G(d) | 295.62 | PCM/6-31G(2d,p) | 297.32 |
| PCM/6-31+G(d) | 304.62 | PCM/6-31+G(2d,p) | 307.1 |
| PCM/6-31++G(d) | 304.64 | PCM/6-31++G(2d,p) | 307.13 |
| PCM/6-311G(d) | 292.02 | PCM/6-311G(2d,p) | 302.16 |
| PCM/6-311+G(d) | 305.74 | PCM/6-311+G(2d,p) | 308.01 |
| PCM/6-311++G(d) | 305.77 | PCM/6-311++G(2d,p) | 308.04 |

**(c)**

| Orbital basis set | Wavelength (nm) | Orbital basis set | Wavelength (nm) |
| --- | --- | --- | --- |
| 6-31G(d) | 633.38 | 6-31G(2d,p) | 641.1 |
| 6-31+G(d) | 651.43 | 6-31+G(2d,p) | 658.03 |
| 6-31++G(d) | 651.51 | 6-31++G(2d,p) | 658.13 |
| 6-311G(d) | 648.28 | 6-311G(2d,p) | 652.26 |
| 6-311+G(d) | 655.69 | 6-311+G(2d,p) | 660.15 |
| 6-311++G(d) | 655.75 | 6-311++G(2d,p) | 660.22 |
| PCM/6-31G(d) | 652.19 | PCM/6-31G(2d,p) | 660.09 |
| PCM/6-31+G(d) | 671.99 | PCM/6-31+G(2d,p) | 678.68 |
| PCM/6-31++G(d) | 672.06 | PCM/6-31++G(2d,p) | 678.79 |
| PCM/6-311G(d) | 668.62 | PCM/6-311G(2d,p) | 672.8 |
| PCM/6-311+G(d) | 676.83 | PCM/6-311+G(2d,p) | 681.16 |
| PCM/6-311++G(d) | 676.88 | PCM/6-311++G(2d,p) | 681.22 |

**Table S1.** Longest wavelength at the peak of the UV/vis spectra from the TDDFT calculation. (a) Functional test. (b) Open ring isomer (experimental value: 286 nm)^1^ with B3LYP functional. (c) Closed ring isomer (experimental value: 600nm)^1^ with B3LYP functional..

**2. System dependency on active space and orbital basis in CASSCF calculation**

Vertical excitation energy (from ground- to first excited state) of each isomer was calculated according to several active spaces and orbital basis set. Active space was referred from Woodward-Hoffmann rules^4^. Our system showed weak dependency on active space and orbital basis as shown in Table S2. Thus, optimization of the conical intersection was performed with CAS(6,6)/6-31G to reduce its massive computational cost, and potential energy surface scan near the branching point was conducted with CAS(10,10)/6-31G(d).

| Active space/  orbital basis | CAS(6,6)/  STO-3G | CAS(6,6)/  6-31G | CAS(6,6)/  6-31G(d) | CAS(10,10)/  STO-3G | CAS(10,10)/  6-31G | CAS(10,10)/  6-31G(d) | CAS(14,14)/  STO-3G | CAS(14,14)/  6-31G | CAS(14,14)/  6-31G(d) |
| --- | --- | --- | --- | --- | --- | --- | --- | --- | --- |
| **Excitation energy of open ring isomer (eV)** | **5.97** | **5.98** | **5.97** | **6.14** | **6.12** | **6.05** | **6.26** | **6.12** | **5.81** |
| **Excitation energy of closed ring isomer (eV)** | **3.91** | **3.96** | **4.08** | **3.92** | **3.94** | **4.01** | **4.06** | **4.17** | **4.26** |

**Table S2.** Vertical excitation energy from ground- to first excited state by CASSCF calculation. (Molecular geometry: geometry optimized structure in ground state by CASSCF with each active space and orbital basis)

**3. Details of no-crossing PESs of first- and second excited state near the CI**

Masunov *et al.* pointed out the PES crossing between the first- and second excited state using a posteriori Tamm-Dancoff approximation and slater transition state method.^5^ Although they focused on different derivatives of diarylethene from ours, validation of no-crossing between the first- and second excited PES was still needed.

To obtain more accurate second excited PES, EOM-CCSD method^6^ which included double excitation operator different from the TD-DFT and CASSCF was applied. Two-dimensional excited PESs were obtained by EOM-CCSD/6-31G(d) calculation, and ground state PES was calculated by B3LYP/6-31G(d) level of theory in Figure S2. The PES scanning was conducted by the same procedure in Figure 2a. As a result, there was no-crossing between the first- and second excited state, and it convincingly demonstrated the reliability of our calculation in Figure 2. Because of the high computational cost, EOM-CCSD method was only employed in the validation of PES no-crossing.


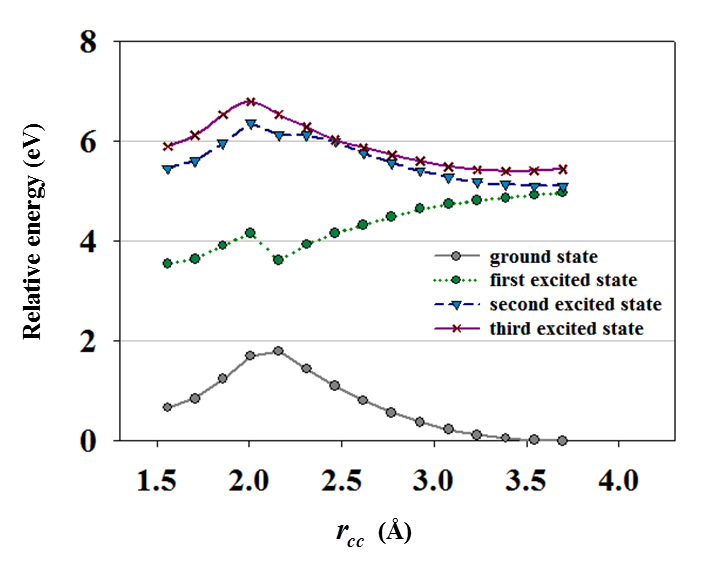


**Figure S2.** Two-dimensional PESs performed by EOM-CCSD method.

**4. Details of temperature dependency of different derivatives**

In Figure 4c, temperature dependency of the statistical model was examined under pulsed visible laser irradiation condition, and showed very similar profile comparing with that of experiment (Ishibashi et al.)^7^. However, they concerned different derivative with ours (our system (DTA): 1,2-bis(2-ethyl-5-phenyl-3-thienyl)perfluorocyclopentene, the one given in Ishibashi et al. (BT)^7^: 1,2-bis(2-methyl-3-benzothienyl)perfluorocyclopentene), and discussion about the difference in molecular structure was needed considering spectroscopic characteristics depending on each structure^8^. Therefore, we conducted non-radiative transition simulation to verify their similar dependency profiles. Population transition path in the simulation was restricted from state 3 to 4 (Figure 1c) where energy barrier on first excited PES exclusively caused the temperature dependency. The procedure was divided into two parts: (a) Characterization of photo-switching property, (b) and Population evolution of non-radiative transition.

**4.1. Characterization of photo-switching property**

Photo-switching property (vibrational relaxation rate constant *k**_34_ and activation energy *ΔE**_34_ from the state 3 to 4) describing the nature of each derivative were obtained as an approximated value from two-dimensional PES to avoid high computational cost of three-dimensional PES scan. Thus, the properties of DTA were derived from Figure 2a and Table 1, and two-dimensional PES scan (Figure S3) was employed to get the ones of BT. Computational details followed the same manner with Figure 2a (geometry optimization with frozen reaction coordinate, vertical excitation with TD-DFT, B3LYP/6-31G(d) level of theory).


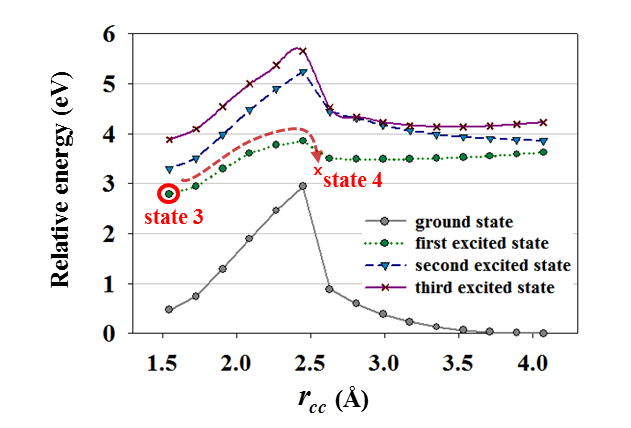


**Figure S3.** Two dimensional PES of BT by geometry optimization and TD-DFT calculation.

Through the two-dimensional PES and frequency calculation combined with transition state theory, the photo-switching properties were calculated (DTA: *k**_34_ (867.20 cm^-1^), *ΔE**_34_ (0.586 eV)/ BT: *k**_34_ (930.16 cm^-1^), *ΔE**_34_ (1.062 eV)), and substituted to density matrices.

**4.2. Population evolution of non-radiative transition**

Based on the statistical photoisomerization modeling section, density matrix formalism of non-radiative transition from state 3 to 4 was constructed as shown in eqns S1 and S2 ($\rho_{3}$: population of state 3, $\rho_{4}$: population of state 4).

$\frac{d\rho_{3}}{dt}=-{k^{*}}_{34}e^{\frac{-\Delta{E^{*}}_{34}}{k_{B}T}}\rho_{3}$ (S1) $\frac{d\rho_{4}}{dt}={k^{*}}_{34}e^{\frac{-\Delta{E^{*}}_{34}}{k_{B}T}}\rho_{3}$ (S2)

Other variables are: the constants *k_B_* and T are the Boltzmann constant and temperature in degrees Kelvin, respectively. *k**_34_ is the vibrational relaxation rate constant from state 3 to 4, and *ΔE**_34_ is the activation energy along state 3 to 4.

Applying the obtained properties (*k**_34_ and *ΔE**_34_) into the density matrices (eqns S1 and S2), non-radiative population evolution from state 3 to 4 was performed, and attained transition rate (state 3 → 4) which was normalized by each value at 343 K as shown in Figure S4. It showed very similar dependency profile between two derivatives, DTA and BT, according to the temperature (253-343 K), and verified the reliability of analogous temperature dependency of each molecule in Figure 4c.


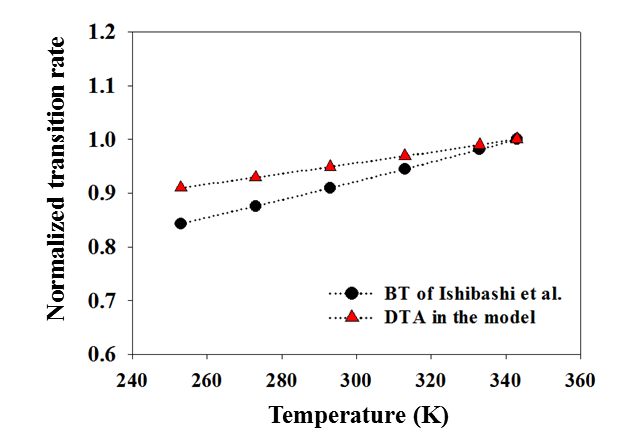


**Figure S4.** Temperature dependency of transition rate (state 3 → 4) of each derivative. BT is 1,2-bis(2-methyl-3-benzotheinyl)perfluorocyclopentene, and DTA indicates 1,2-bis(2-ethyl-5-phenyl-3-thienyl)perfluorocyclo

pentene.

**5. Primary stimuli and molecular design parameter for rapid isomerization**

We have provided the following two new important studies for controlling the isomerization rate through the sole statistical isomerization model. The contents are separated into two parts: **5.1.** effect of external stimuli (phenomenological findings), **5.2.** and lagging physics on population transition (discussion).

**5.1 Effect of external stimuli (phenomenological findings)**

The statistical photoisomerization model (eqn 1-3) reflects external stimulus parameters such as the type of light source, intensity and polarization angle of light, and temperature. Thus, the population data from the model is affected by the above parameters, and its dependency profile is clearly found on photoisomerization simulation data (Figure S5).

The light intensity (*n_uv_* and *n_vis_*) applied in the model is in the range of 2.0*10^26^-2.0*10^27^ photons/s (UV: 20.517-205.17 MW/cm^2^, vis: 3.4794-34.794 MW/cm^2^). Other external stimulus factors are (*θ_UV_*, *θ_vis_*: angle between polarized light and electric transition dipole moment)= 0-60° and *T*= 270-360 K. Under these simulation conditions, the effects of the light irradiation parameters for the cyclization and cycloreversion were examined (Figure S5). The effects were characterized by the reaction time, which was measured when 99% of the reactant population went to the product state. For the temperature dependence of the cycloreversion, the results were directly represented in terms of population (Figure S6).

Figure S5a indicates the cyclization reaction time according to *n_uv_* and *θ_UV_*. Temperature was not considered as an external stimulus parameter for the cyclization, because the transition path does not include an energy barrier causing temperature dependence. Strong UV intensity stimulates a fast population transition overall. In the vicinity of the faint UV region, isomerization speed decreases rapidly as *θ_UV_* increases. The reaction time shows weak dependency about the angle with irradiation from the strong UV region. These tendencies are ascribed to the interaction energy term *V_mj_*, the source of the population transition, which consists of the *n_uv_* and *θ_UV_* parameters. Increasing the UV intensity augments the interaction energy and the angle increase reduces the energy as a cosine term (eqn 8). Therefore, the variation of the interaction energy affects the population transition, and the extent of the negative influence by the angle has insignificant effect under the strong UV irradiation compared to the weak intensity case.


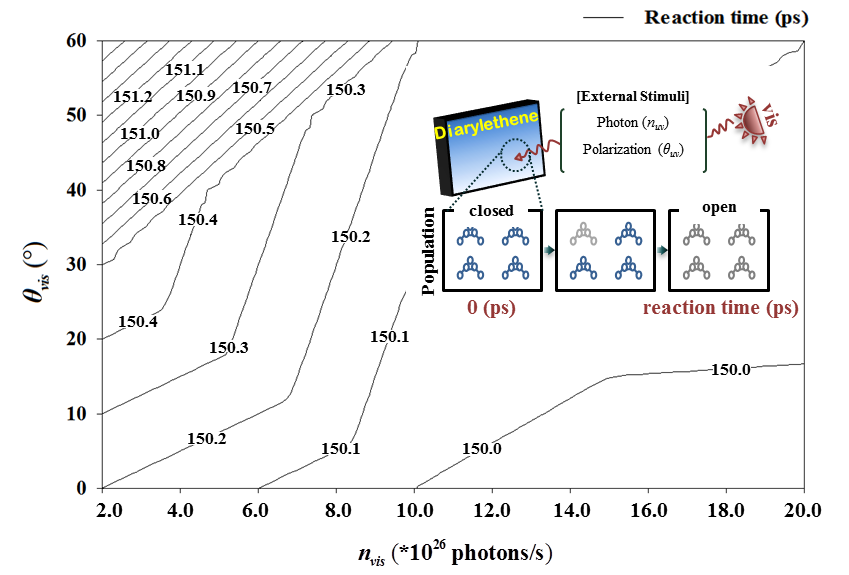

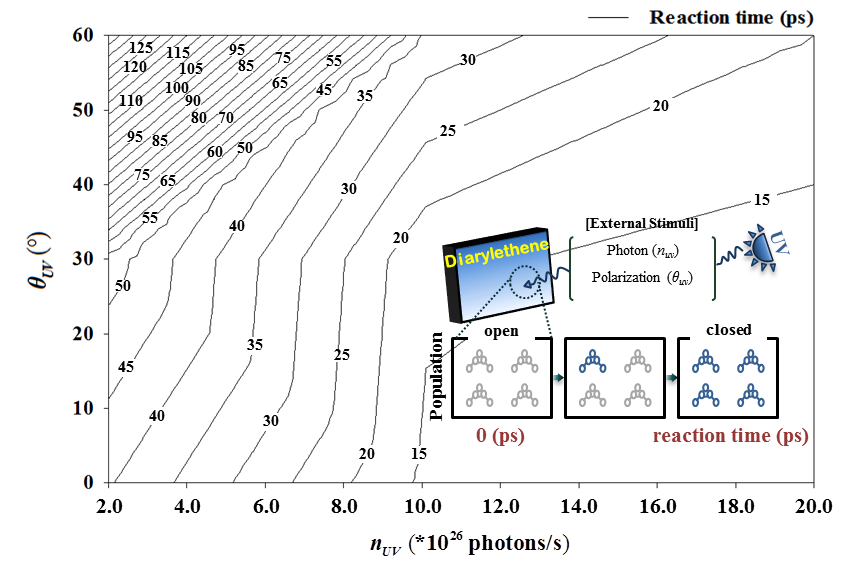


**(b)**

**(a)**

**Figure S5.** (a) Cyclization reaction time according to *n_uv_* and *θ_UV_*. (b) Cycloreversion reaction time according to *n_vis_* and *θ_vis_* at 300 K. Insets indicate schematic figure of photoisomerization of isomers. (*n_uv_* and *n_vis_* are the number of photons per second. *θ_UV_* and *θ_vis_* indicate light polarization condition.)


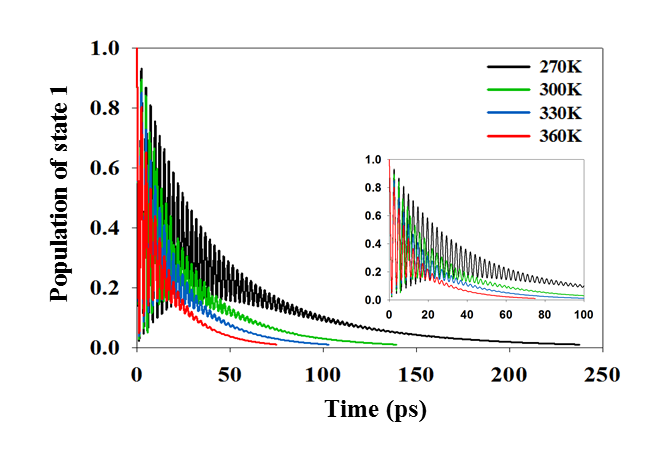

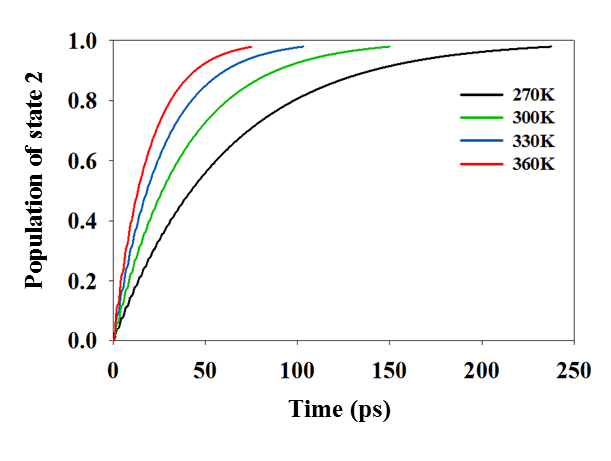


**(b)**

**(a)**

**Figure S6.** Temperature dependence of cycloreversion (*n_vis_* = 1.0*10^27^ photons/s, and *θ_vis_* = 0). (a) Population of the ground state of the open ring isomer. (b) Population of the ground state of the closed ring isomer. Early time data (~100 ps) is shown in inset. (*n_vis_* is the number of photons per second. *θ_vis_* indicates light polarization condition.)

The cycloreversion reaction time is plotted with *n_vis_* and *θ_vis_* at 300 K, as shown in Figure S5b. The light intensity and the angle have an influence similar to the case of cyclization. However, the reaction time is longer for cycloreversion than it is for cyclization, and the range of the changes in magnitude is narrower. Temperature dependence of the cycloreversion was also investigated at *n_vis_* = 1.0*10^27^ photons/s, and *θ_vis_* = 0 as shown in Figure S6. The population transfers from the ground closed-ring to ground open-ring state, and the transfer becomes faster as the temperature increases, which corresponds to the experiment^7^ of Ishibashi et al. The longer reaction time and temperature dependence of the cycloreversion are caused by the influence of the activation energy during the transition. Among the transition processes (excitation, spontaneous decay, vibrational relaxation), the energy barrier of the first excited PES slows down the vibrational relaxation rate as reflected by the influence of the Boltzmann factor on the characteristics of cycloreversion.

**5.2 Lagging physics on population transition (discussion)**

Beyond the phenomenological findings, the underlying physics are discussed by investigating the population data of each state. The population data in Figure 3 shows what extent the population consumes a certain period to pass through the state, so called lagging physics. The lagging effect is measured as population lag constant by integrating the population-time curve, and faster population transition at a given state will have a smaller


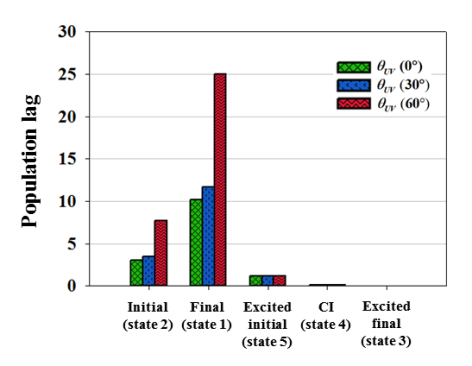

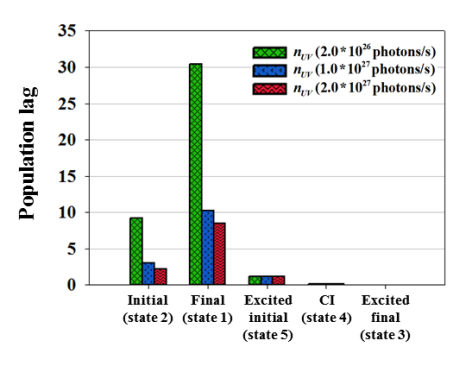


**(b)**

**(a)**


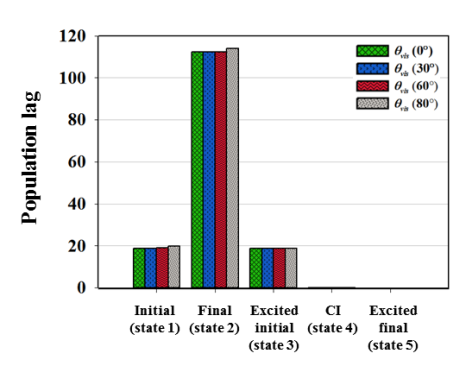

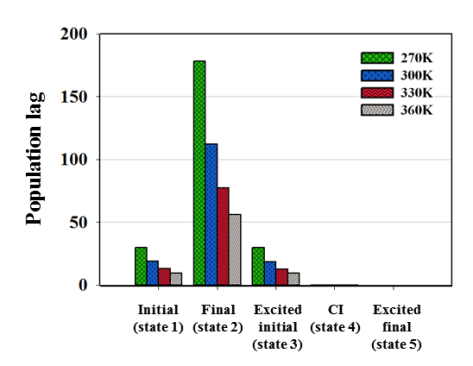

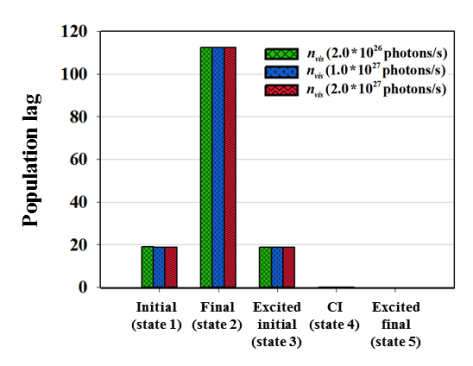


**(d)**

**(e)**

**(c)**

**Figure S7.** Population lag value of each molecular state (with state number from calculations in parentheses). (a) Cyclization according to the *n_uv_* (*θ_UV_* = 0). (b) Cyclization according to the *θ_UV_* (*n_uv_* = 1.0*10^27^ photons/s). (c) Cycloreversion according to the *n_vis_* (*θ_vis_* = 0, *T* = 300K). (d) Cycloreversion according to the *θ_vis_* (*n_vis_* = 1.0*10^27^ photons/s, *T* = 300K). (e) Cycloreversion according to the temperature (*n_vis_* = 1.0*10^27^ photons/s, and *θ_vis_* = 0). (*n_uv_* and *n_vis_* are the number of photons per second *θ_UV_* and *θ_vis_* indicate light polarization condition.)

integrated value. Considering the population transition path in Figure 1c, lagging population at the initial and intermediate (excited initial and CI) states dominates the overall rate of transition.

For the cyclization, the population lag value for each state is normalized according to the *n_uv_* and *θ_UV_* as shown in Figure S7a and S7b. Among the dominant states for the transition rate, most of the population is stagnant at the initial state (state 2), but there is a small portion populating the intermediate states 4 and 5. The lag value at the initial state is highly dependent on the light intensity and the angle. It can be interpreted that UV light with strong intensity and *θ_UV_* ~ 0˚ directly helps the population to escape from state 2 (corresponding to the excitation) which is a primary obstacle for the transition, and causes fast photoisomerization (Figure S5a).

Through the integration of the population-time curve (e.g. Figure 3c and 3d) of cycloreversion, the lag value is obtained, as shown in Figure S7c-e. The lag value is normalized to the same scale as the cyclization case according to the *n_vis_*, *θ_vis_*, and *T*. Two different things are learned from a comparison with the cyclization. First, the population lag value is higher for the cycloreversion than the cyclization, which leads to longer reaction time. Second, both the initial state 1 and the excited initial state 3 affect most of the delaying phenomenon equally. The equivalent influences of state 1 and 3 originate from the oscillation profile mentioned in Figure 3c.

Thus, it is important to discuss the effect of light irradiation and the temperature on state 1 and state 3. In Figure S7c and S7d, the light irradiation has an insignificant influence on the lag at states 1 and 3. According to the above discussion regarding Figure S7a and S7b, the light property induces the escape (excitation) from the initial state, but the population lag at state 1 shows less dependence. There is an energy barrier (0.1073 eV) on the first excited PES during the cycloreversion, and this is different from the cyclization in that it causes significant lagging of the population transfer from state 3 to 4. This barrier returns most of the excited population at state 3 to state 1 which is reflected by oscillation profile as shown in Figure 3c. Therefore, the energy barrier reduces the influences of light irradiation, and temperature which helps the population overcome the energy barrier becomes the dominant external stimulus parameter. With higher temperature, population lag at state 3 decreases along with state 1 (Figure S7e), and makes the transition fast.

In addition, the population lag data also provides a guideline to design the molecule performing rapid isomerization. Initial state seriously hinders the population transition of cyclization (Figure S7a and b), and large electric transition dipole moment (*d_25_* in Table 2) is a design parameter to enhance the isomerization rate in that interaction energy (*V_mj_* in eqn 8) induces the population to escape from the initial state as a driving source of the excitation. In case of cycloreversion, excited initial state occupies large part of the lagging phenomenon (Figure S7c-e). Thus, low energy barrier (*ΔE_34_* in Table 2) and large vibrational relaxation rate constant (*k_34_* in Table 2) on first excited PES are design parameters which relieve the stagnation on the excited initial state. This theoretical prediction about predominant external stimuli (light irradiation conditions for cyclization, and temperature for cycloreversion) and molecular design parameters (*d_25_* for cyclization/ *ΔE_34_* and *k_34_* for cycloreversion) are crucial findings in the consideration of their influences on isomerization rate.

**6. Generating metastable excited closed ring state**

It is generally known that population of excited state of closed ring isomer decays to ground open ring isomer via conical intersection, and converges to zero during cycloreversion.^9,10^ However, we predicted that the trivial phenomenon, decaying excited closed ring state during cycloreversion, would change under simultaneous irradiation of ultraviolet (UV) and visible (vis) light.

Using the established statistical photoisomerization model, population evolution of each state was calculated under simultaneous irradiation on open ring diarylethene (Figure S8a). The light intensity was 2.0*10^26^ photons/s (*n_uv_* ~ 20.517 MW/cm^2^) for UV (350.58 nm) and 2.0*10^25^ photons/s (*n_vis_* ~ 347.940 kW/cm^2^) for vis (633.38 nm) irradiation. Other stimulus factors are *θ_UV_* and *θ_vis_* (angle between polarized light and electric transition dipole moment: 0˚) and *T* (temperature: 300 K).

In usual cycloreversion as shown in Figure S8b, vis light photons stimulate excitation of ground closed ring state (state 1), and energy barrier on first excited potential energy surface (see Figure 1c and 2) returns most of the excited population (state 3). Thus, few of population in state 3 travels to ground open ring state through conical intersection (CI), and it causes oscillation profile of state 1 and 3 under continuous irradiation. Its amplitude converges to zero as the population of state1 consequently transfers to state 2.

On the other hand, metastable excited closed ring (state 3) is generated under simultaneous irradiation of UV and vis photons on open ring isomer (Figure S8a), and its stable profile originates from that the population continuously flows into ground closed ring state (state 1) by synchronous cyclization process, caused by UV photons. As a result, the population becomes equilibrium state (population transition still proceeds among multiple states), and diarylethene contains much more population in excited closed ring state than that of the usual cycloreversion case.

We deduced that this phenomenon would enhance fluorescence performance of 1,2-bis(2-methyl-3-benzothienyl)perfluorocyclopentene (BT). Shim et al. reported that closed ring isomer of BT emitted weak fluorescence at 630 nm^11^, and the photon emission (fluorescence) indicated energy dissipation between excited- and ground state when excited electrons spontaneously decayed to the ground state. Its emission rate is proportional to population of excited state^12^, and simultaneous irradiation, generating more population of excited closed ring state than that of normal cycloreversion, would improve fluorescence performance of BT at 630 nm in that BT had qualitatively identical potential energy surface to our system (see Figure 2 and S3).

**(a)**
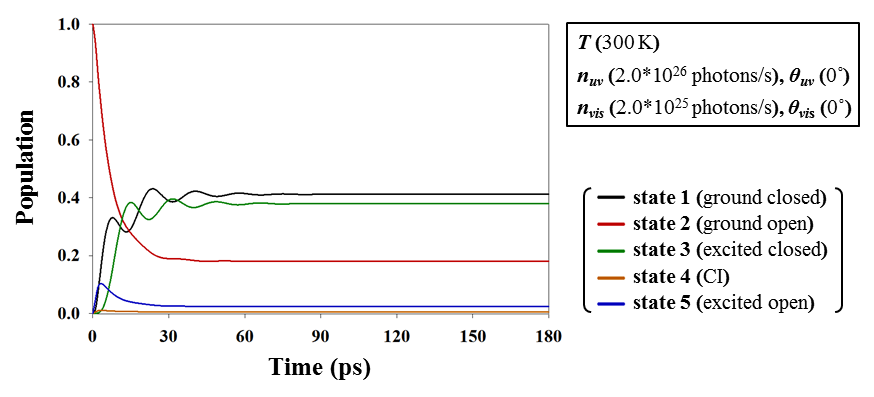


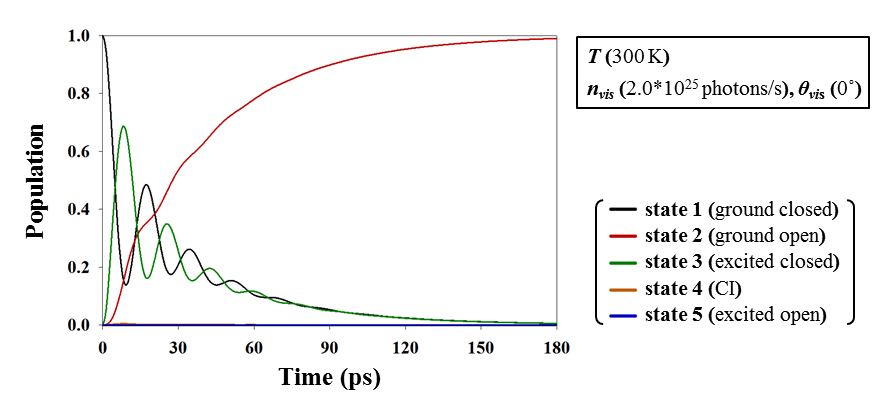
**(b)**

**Figure S8.** Population evolution under (a) simultaneous irradiation of UV and vis light, and (b) visible light only. (*n_uv_* and *n_vis_* are the number of photons. *θ_UV_* and *θ_vis_* indicate light polarization condition. *T* is temperature)

On the other hand, metastable excited closed ring (state 3) is generated under simultaneous irradiation of UV

**7. Details of light penetration coupling methodology**

Below figures explain further information of methodology section in the manuscript.


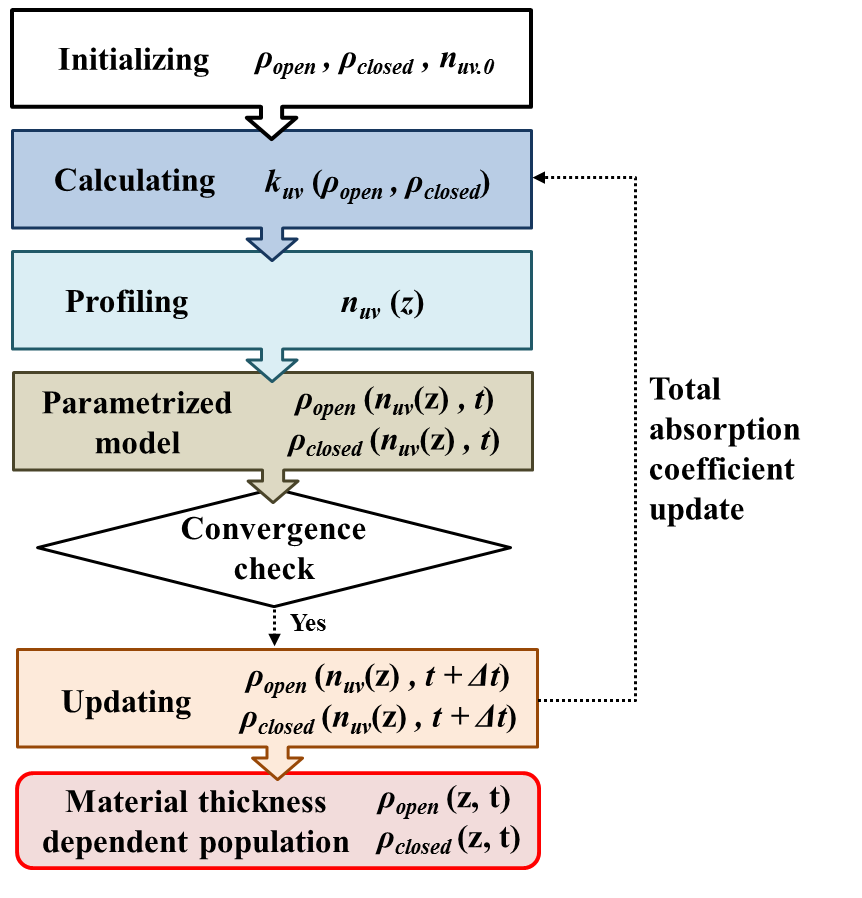


**Figure S9.** Iteration algorithm of light penetration-coupled statistical photoisomerization model. (*n_uv.0_* is the number of UV photons per second which are irradiated on the material surface)


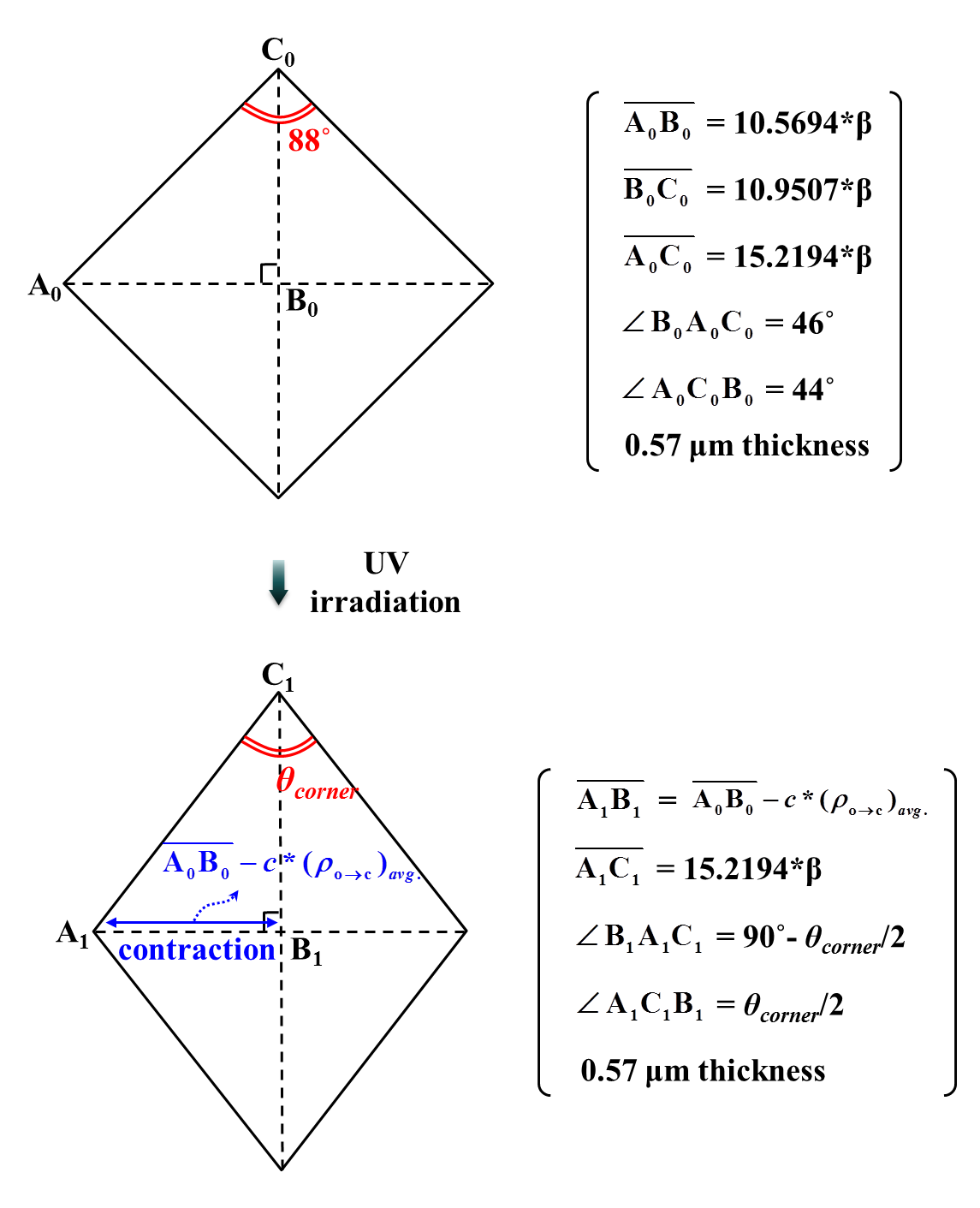


**Figure S10.** In-plane shear deformation of 1,2-bis(2-ethyl-5-phenyl-3-thienyl)perfluorocyclopenetene single crystal.^13^ Insets indicate size and angle of the sample. β is normalization constant.

**8. Details of density matrix equations**

Below table indicates derived terms which are substituted to density matrix equations in eqn 1 and 2 in the manuscript.

| Spontaneous decay term (sec-1) | | | | | | | | | |
| --- | --- | --- | --- | --- | --- | --- | --- | --- | --- |
| **Γ_33_** | | **Γ_55_** | | | **Γ_31_** | | | **Γ_52_** | |
| 0.0138 | | 0.0044 | | | 0.0069 | | | 0.0022 | |
| **Vibrational relaxation term (cm-1)** | | | | | | | | | |
| **Κ_11_** | **Κ_22_** | | **Κ_33_ / *f_boltzmann_*** | **Κ_44_** | | **Κ_55_** | **Κ_31_ / *f_boltzmann_*** | | **Κ_52_** |
| 271.96 | 271.96 | | 221.14805 | 543.92 | | 55.17 | 110.574025 | | 27.585 |

**Table S3.** Component terms of density matrix equations (each variable listed in eqns 1,2)

**9. Details of reliability test of statistical photoisomerization model**

Below table indicates calculated time constants of the product with visible pulse laser irradiation.

| Temperature (K) | 253 | 273 | 293 | 313 | 333 | 343 |
| --- | --- | --- | --- | --- | --- | --- |
| **Time constant (ps)** | **20.636** | **14.414** | **10.565** | **8.016** | **6.321** | **5.679** |

**Table S4.** Time constants of population of state 3 under vis pulse laser irradiation.

**10. Application of polarization effect to the light penetration-coupled photoisomerization model**

To reflect the polarization condition, parametrized photoisomerization model (eqns S3-S5) was derived from the density matrix formulation (eqns 1-3).

$\rho_{open}=e^{-\left[ \left( {10}^{\alpha}-1 \right)*t \right]}$ (S3) $\rho_{closed}=1-\rho_{open}$ (S4)

$\alpha=10.95*e^{-\left[ \frac{\log\left( n_{uv}\left( \frac{\pi\cos\theta}{2} \right)^{2}+1 \right)-28.3}{3.65} \right]^{2}}$ (S5)

Population of ground open- and closed ring isomer are represented as *ρ_open_* (*ρ_22_* ~ in eqn 1) and *ρ_closed_* (*ρ_11_* ~ in eqn 1), respectively. *n_uv_* indicates the number of ultraviolet photons (at 350.58 nm) per second, and *θ* is the included angle between the linearly-polarized light and electric transition dipole moment of the molecule. Each transition dipole moment of open ring isomers in the unit cell ([*d_25_*] _molecule 1_ - [*d_25_*] _molecule 4_ in Figure R1) was obtained by first principle calculation (geometry optimization, B3LYP/6-31G(d) level of theory), and used to calculate *θ* (in eqn. R3) of each isomer under linearly-polarized light irradiation. As the last part of the methodology, the light penetration coupling (eqns 15 and 16) was conducted with the parametric model (eqn S3-S5), and iteration algorithm in Figure S9 was employed to investigate the isomerization progress along the light penetrating direction.

By rotating the crystal system (modulating *θ_rot_* of Figure S11a) under 60 mW/cm^2^ (5.85*10^17^ photons/s) intensity of polarized ultraviolet light (350.58 nm), cyclization conversion ratio within the material thickness (0.57 μm) was predicted after 200 seconds of elapsed time, and its magnitude (0.4-22.6 % conversion) was normalized for convenient comparison as shown in Figure S11b.

When the material is rotated by 90° and 270°, the open ring isomers are easily converted to the closed ring state, but on the other hand, poor conversion ratio is found near the 0° and 180° of *θ_rot_*. Its anisotropic feature according to the polarization condition was also followed by the

**
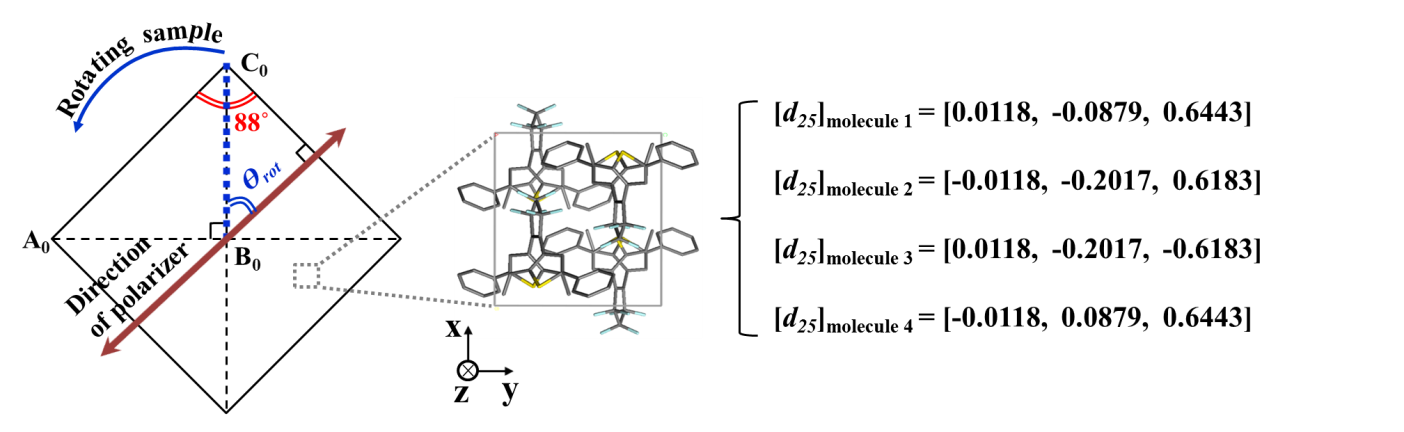
(a)**


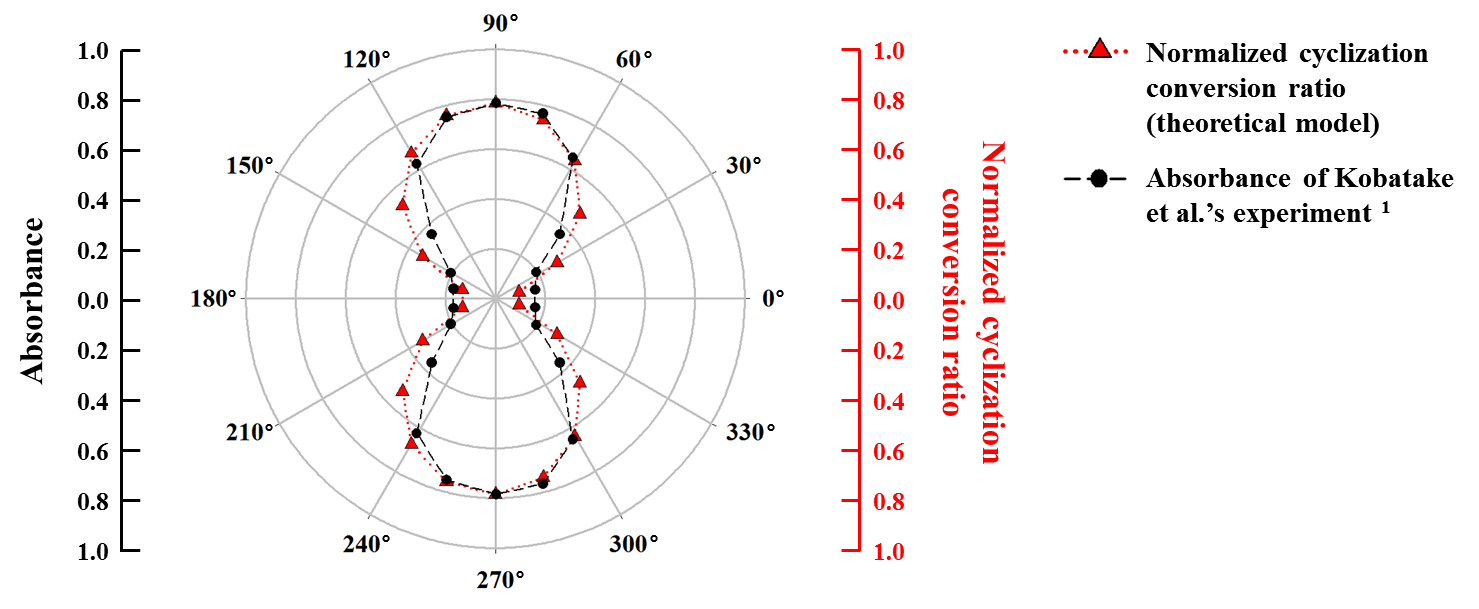


**(b)**

**Figure S11.** (a) Schematic illustration of single crystal with linearly-polarized light source (sample size: same as the values of crystal in Figure S10, molecular packing in the unit cell: referred from Kobatake et al.’s paper^13^, *d_25_*: electric transition dipole moment in atomic unit). (b) Polar plot of normalized cyclization conversion ratio under various polarization conditions (wavelength: 350.58 nm, intensity: 60 mW/cm^2^, polarization: 0-360° of *θ_rot_*). Experimental comparison (angular dependency of absorbance) is referred from Kobatake et al.’s paper^1^.

absorbance of reference experiment^1^. The qualitative similarity of the angular dependency validates the reliability of polarization effect embedded-theoretical model, because the conversion ratio should be proportional to the absorbance in that a huge number of absorbed photons would lead large amount of isomerization progress. In addition, Kobatake et al.^1^ reported that the crystal turned blue at 90° of rotation under polarized ultraviolet light source and its color almost disappeared as modulating *θ_rot_* to 0° (open ring isomer: colorless, closed ring isomer: blue), and this optical behavior also strengthened the reliability of the model (high conversion ratio = closed ring population ↑ = blue color ↑).

As a result, we successfully applied the linearly-polarized light source to the original model, and analysis of mechanical behavior of diarylethene single crystal under various polarization conditions will be discussed in the future work.

**References**

1. Kobatake, S., Shibata, K., Uchida, K. & Irie, M. Photochromism of 1,2-Bis(2-ethyl-5-phenyl-3-thienyl)perfluorocyclopentene in a Single-Crystalline Phase. Conrotatory Thermal Cycloreversion of the Closed-Ring Isomer. *J. Am. Chem. Soc.* **122**, 12135-12141 (2000).

2. Staykov, A. *et al.* Electrochemical and Photochemical Cyclization and Cycloreversion of Diarylethenes and Diarylethene-Capped Sexithiophene Wires. *ACS Nano* **5**, 1165-1178 (2011).

3. Staykov, A. & Yoshizawa, K. Photochemical Reversibility of Ring-Closing and Ring-Opening Reactions in Diarylperfluorocyclopentenes. *J. Phys. Chem. C* **113**, 3826-3834 (2009).

4 Woodward, R. & Hoffmann, R. *The conservation of Orbital Symmetry*, Verlag Chemie, (1970).

5 Mikhailov, I. A., Belfield, K. D. & Masunov, A. E. DFT-Based Methods in the Design of Two-Photon Operated Molecular Switches. *J. Phys. Chem. A* **113**, 7080-7089 (2009).

6. Stanton, J. F. & Bartlett, R. J. The Equation of Motion Coupled‐Cluster Method. A Systematic Biorthogonal Approach to Molecular Excitation Energies, Transition Probabilities, and Excited State Properties. *J. Chem. Phys.* **98**, 7029-7039 (1993).

7. Ishibashi, Y. *et al.* Femtosecond Laser Photolysis Studies on Temperature Dependence of Cyclization and Cycloreversion Reactions of a Photochromic Diarylethene Derivative. *J. Phys. Chem. C* **116**, 4862-4869 (2012).

8. Sumi, T. *et al.* Photoirradiation wavelength dependence of cycloreversion quantum yields of diarylethenes. *Chem. Commun.* **50**, 3928-3930 (2014).

9. Wiebeler, C., Bader, C. A., Meier, C. & Schumacher, S. Optical Spectrum, Perceived Color, Refractive Index, and Non-adiabatic Dynamics of the Photochromic Diarylethene CMTE. *Phys. Chem. Chem. Phys.* **16**, 14531-14538 (2014).

10. Wiebeler, C. & Schumacher, S. Quantum Yields and Reaction Times of Photochromic Diarylethenes: Nonadiabatic Ab Initio Molecular Dynamics for Normal- and Inverse-Type. *J. Phys. Chem. A* **118**, 7816-7823 (2014).

11. Shim, S. *et al.* Ring Opening Dynamics of a Photochromic Diarylethene Derivative in Solution. *J. Phys. Chem. A* **107**, 8106-8110 (2003).

12. Kraĭnov, V. P., Reiss, H. & Smirnov, B. M. *Radiative processes in atomic physics*, Wiley, (1997).

13. Kobatake, S. *et al.* Rapid and Reversible Shape Changes of Molecular Crystals on Photoirradiation. *Nature* **446**, 778-781 (2007).

14. Morimoto, M. & Irie, M. A Diarylethene Cocrystal that Converts Light into Mechanical Work. *J. Am. Chem. Soc.* **132**, 14172 (2010).
